# A Conserved Long Intergenic Non-coding RNA Containing snoRNA Sequences, *lncCOBRA1*, Affects Arabidopsis Germination and Development

**DOI:** 10.3389/fpls.2022.906603

**Published:** 2022-05-25

**Authors:** Marianne C. Kramer, Hee Jong Kim, Kyle R. Palos, Benjamin A. Garcia, Eric Lyons, Mark A. Beilstein, Andrew D. L. Nelson, Brian D. Gregory

**Affiliations:** ^1^Department of Biology, University of Pennsylvania, Philadelphia, PA, United States; ^2^Cell and Molecular Biology Graduate Group, Perelman School of Medicine, University of Pennsylvania, Philadelphia, PA, United States; ^3^Department of Biochemistry and Biophysics, Perelman School of Medicine, University of Pennsylvania, Philadelphia, PA, United States; ^4^Epigenetics Institute, Perelman School of Medicine, University of Pennsylvania, Philadelphia, PA, United States; ^5^Biochemistry and Molecular Biophysics Graduate Group, University of Pennsylvania, Philadelphia, PA, United States; ^6^School of Plant Sciences, University of Arizona, Tucson, AZ, United States; ^7^CyVerse Inc., Tucson, AZ, United States; ^8^Boyce Thompson Institute, Cornell University, Ithaca, NY, United States

**Keywords:** long non-coding RNA, lincRNA, long intergenic non-coding RNA, snoRNA, RNA binding protein, RNA, *Arabidopsis thaliana*

## Abstract

Long non-coding RNAs (lncRNAs) are an increasingly studied group of non-protein coding transcripts with a wide variety of molecular functions gaining attention for their roles in numerous biological processes. Nearly 6,000 lncRNAs have been identified in *Arabidopsis thaliana* but many have yet to be studied. Here, we examine a class of previously uncharacterized lncRNAs termed *CONSERVED IN* BRASSICA RAPA (*lncCOBRA*) transcripts that were previously identified for their high level of sequence conservation in the related crop species *Brassica rapa*, their nuclear-localization and protein-bound nature. In particular, we focus on *lncCOBRA1* and demonstrate that its abundance is highly tissue and developmental specific, with particularly high levels early in germination. *lncCOBRA1* contains two snoRNAs domains within it, making it the first sno-lincRNA example in a non-mammalian system. However, we find that it is processed differently than its mammalian counterparts. We further show that plants lacking *lncCOBRA1* display patterns of delayed germination and are overall smaller than wild-type plants. Lastly, we identify the proteins that interact with *lncCOBRA1* and propose a novel mechanism of lincRNA action in which it may act as a scaffold with the RACK1A protein to regulate germination and development, possibly through a role in ribosome biogenesis.

## Introduction

Long non-coding RNAs (lncRNAs) are transcripts defined as greater than 200 nucleotides (nt) that lack or have an open reading frame less than 100 amino acids (Liu et al., 2012). Transcriptome-wide studies have demonstrated that lncRNAs are often expressed in a context-specific manner, a characteristic believed to facilitate some of their known functions in modulating gene expression, mRNA splicing, and translation (Quinn and Chang, 2016). The function of lncRNAs is highly dependent on their subcellular location. Nuclear lncRNAs often serve key roles in regulating gene expression, either in *cis* (where the lncRNA interacts with neighboring genes to regulate their expression) or in *trans* (where the lncRNA interacts with distant genes to regulate their expression). lncRNAs can also bind and sequester proteins, such as proteins involved in chromatin stability and splicing factors, from their target chromosomal regions, thereby affecting gene expression (Yin et al., 2012; Lee et al., 2016).

In plants, lncRNAs are implicated in numerous biological mechanisms with demonstrated functions in flowering, organogenesis, photomorphogenesis, reproduction, and abiotic/biotic stress responses (reviewed in Wang and Chekanova, 2017). Most research has focused on the intergenic class of lncRNAs (lincRNAs) (Mattick and Rinn, 2015), as historically it has been easier to discern their transcriptional origins relative to other lncRNAs that overlap protein-coding genes [e.g., natural antisense transcripts (NATs)]. In plants, detailed annotation and functional efforts have led to the identification of several lincRNAs with characterized functions in regulation of auxin signaling outputs (Ariel et al., 2014), response to abiotic and biotic stressors (Wang et al., 2014; Qin et al., 2017; Seo et al., 2017), flower timing (Swiezewski et al., 2009), and response to phosphate starvation (Franco-Zorrilla et al., 2007).

While most Pol II transcribed lincRNAs are 5′ capped and 3′ polyadenylated, recently a previously uncharacterized group of lncRNAs that lacks one or both of these features has been described (Xing and Chen, 2018). These non-canonical Pol II-dependent lncRNAs have snoRNA sequences at their 5′ and 3′ ends and are referred to as sno-lncRNAs. snoRNAs are 70–200 nt highly structured, nuclear-localized, protein-bound non-coding RNAs that are usually concentrated in the Cajal bodies or nucleolus (Reichow et al., 2007). snoRNAs co-transcriptionally form snoRNA-ribonucleoprotein complexes (snoRNPs) (Kiss, 2001) and function through complementarity with ribosomal RNA (rRNA) sequences to guide rRNA modification to ultimately participate in ribosome subunit maturation. The formation of snoRNPs at the ends of sno-lncRNAs protects the intervening sequence from exonuclease trimming (Yin et al., 2012).

sno-lncRNAs have been identified in humans, rhesus monkeys, and mice (Yin et al., 2012; Zhang et al., 2014; Xing et al., 2017) but have yet to be described in plants. A functional analysis of sno-lncRNAs in humans was recently performed, where SLERT was identified (snoRNA-ended lncRNA enhances pre-ribosomal RNA transcription; Xing et al., 2017). SLERT localizes to the nucleolus in a manner dependent on the two snoRNPs at its ends and functions to promote active transcription of rRNAs (Xing et al., 2017). Thus, sno-lncRNAs represent an interesting class of lncRNAs with evident functions in humans.

Due to their lack of protein-coding capacity, lincRNAs typically display poor sequence conservation among even closely related species (Necsulea et al., 2014; Nelson et al., 2016). lincRNAs with functions defined by structural or sequence-specific interactions with other molecules (e.g., proteins) will likely display higher levels of conservation over lincRNAs that function based on proximity to other genes (e.g., transcription enhancers/repressors). We previously identified lincRNAs in the nuclei from 10-day-old seedlings and found that lincRNAs with RNA binding protein (RBP) binding sites were significantly more likely to be conserved at the sequence-level in *Brassica rapa* than those that lacked protein binding sites (Gosai et al., 2015), suggesting these protein-bound, conserved lincRNAs may be of functional importance in plants.

Here, we assess the function of those nuclear, protein-bound, and conserved lincRNAs that we have termed *CONSERVED IN* BRASSICA RAPA (*lncCOBRA*). We find that the *COBRA* lincRNAs display germination- and developmental-dependent patterns of abundance and, in particular, we focus on *lncCOBRA1* which contains two snoRNA sequences within it, indicating the first evidence of a sno-lincRNA in Arabidopsis. Unlike sno-lncRNAs identified in humans, *lncCOBRA1* is transcribed from an intergenic region, and is transcribed as a longer transcript before processing at its 3′ end to a final length of ∼500–600 nt. We further show that *lncCOBRA1* influences plant germination and growth, as plants lacking *lncCOBRA1* germinate later and are smaller than wild type plants. Lastly, we identify *lncCOBRA1*-interacting proteins, including the scaffold protein RACK1A, and several of its known interactors and hypothesize that *lncCOBRA1* functions with RACK1A to affect ribosome biogenesis.

## Results

### Identification of Conserved, Nuclear, Protein-Bound Long Intergenic Non-coding RNAs

We previously identified 236 nuclear lincRNAs from 10-day-old seedlings, of which 38 contained up to four RNA-binding protein (RBP) interaction sites (Gosai et al., 2015). These protein-bound lincRNAs were significantly more conserved within the related crop species *Brassica rapa* than those lacking RBP binding sites (Supplementary Figure 1A and Table 1; Gosai et al., 2015). Since lincRNAs do not encode proteins, small polymorphisms within the sequence generally have little functional consequence, and thus lincRNAs are generally not well conserved at the sequence level (Ponjavic et al., 2007; Necsulea et al., 2014; Hezroni et al., 2015). Thus, the combination of conservation in *Brassica rapa* and nuclear protein binding suggested that these lincRNAs may have important functions in plant systems and were named *CONSERVED IN BRASSICA RAPA 1-14* (*lncCOBRA1-14*) (Supplementary Figure 1A and Table 1).

**TABLE 1:** Protein-bound lincRNAs from Gosai et al. (2015).

Liu et al., 2012	Araport11	COBRA ID
***AT1NC031460***	***AT1G05913***	lncCOBRA1*
***AT3NC000460***	***AT3G00980***	lncCOBRA2*
***AT3NC020890***	***AT3G03435***	lncCOBRA3*
***AT3NC032690***	***AT3G04775***	lncCOBRA4*
***AT3NC040900***	***AT3G05655***	***lncCOBRA5***
***AT3NC086300***	***AT3G09105***	lncCOBRA6*
***AT4NC002760***	***AT4G03905***	lncCOBRA7*
***AT4NC005680***	***AT4G04565***	***lncCOBRA8***
***AT4NC034360***	***AT4G13575***	***lncCOBRA9***
***AT4NC036680***	***AT4G06235***	lncCOBRA10*
***AT4NC048800***	***AT4G07070***	lncCOBRA11*
***AT5NC020840***	***AT5G02645***	lncCOBRA12*
***AT5NC077900***	***AT5G07325***	***lncCOBRA13***
***AT5NC082220***	***AT5G07745***	***lncCOBRA14***
*AT1NC002820*		
*AT1NC006050*		
*AT1NC020200*		
*AT1NC078930*		
*AT2NC000010*		
*AT2NC003350*		
*AT2NC058570*		
*AT3NC007270*		
*AT3NC007290*		
*AT3NC021940*		
*AT3NC034870*		
*AT3NC040560*		
*AT3NC056191*		
*AT3NC092460*		
*AT4NC046820*		
*AT5NC011780*		
*AT5NC015150*		
*AT5NC015260*		
*AT5NC033040*		
*AT5NC034990*		
*AT5NC087850*		
*AT5NC096690*		
*AT5NC097520*		
*AT5NC101430*		

*Denotes sno-lincRNA. Bold denotes conserved in B. rapa.

**FIGURE 1:**
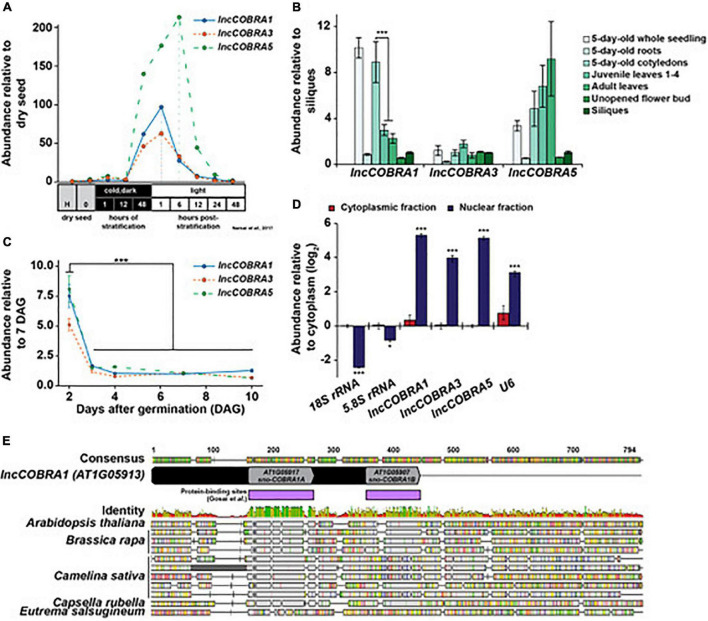
Identification and expression of highly conserved, protein-bound lincRNA, *lncCOBRA1.* **(A)** Abundance of *lncCOBRA1*, *lncCOBRA3*, and *lncCOBRA5* during germination as previously measured by RNA-seq (Narsai et al., 2017). Abundance is relative to dry seed after harvest. Raw values are listed in Supplementary Data Set 3. **(B)** Abundance of *lncCOBRA1*, *lncCOBRA3*, and *lncCOBRA5* in various tissues as measured by qPCR. Abundance is normalized by the geomean of *UBC9* and *UBC10* and is relative to siliques seedlings. *** Denotes *p*-value < 0.001, Wilcoxon *t*-test. **(C)** Abundance of *COBRA1*, *lncCOBRA3*, and *lncCOBRA5* during early seedling development as measured by qPCR. Abundance is normalized by the geomean of *UBC9* and *UBC10* and relative to 7-day-old seedlings. *** Denotes *p*-value < 0.001, Wilcoxon *t*-test. **(D)** Abundance of *lncCOBRA1*, *lncCOBRA3*, and *lncCOBRA5* in nuclear and cytoplasmic fractions as measured by qPCR. Abundance is normalized to *UBC9* and relative to cytoplasmic fraction. *18S rRNA* and *5.8S rRNA* are cytoplasmic positive controls and *U6* is a nuclear positive control. *, *** Denotes *p*-value < 0.05, <0.001, Wilcoxon *t*-test. **(E)** Conservation *lncCOBRA1* in *Brassica rapa, Camelina sativa, Capsella rubella*, and *Eutrema salsugineum.* Conservation was examined using Geneious Prime (Geneious | Bioinformatics Solutions for the Analysis of Molecular Sequence Data, 2019). Protein-binding sites were identified in the nuclei from 10-day-old seedlings in (Gosai et al., 2015). Colors in identity: Green = 100%, green-brown = 30–100%, red < 30% identity.

We selected a set of these *lncCOBRA*s and initiated our search for function by examining their abundance profiles during seed germination, as lincRNAs in several eukaryotic species are essential during development (e.g., *HOTAIR*, *COOLAIR*) (Rinn et al., 2007; Swiezewski et al., 2009; Liu et al., 2012; Sarropoulos et al., 2019). Using a previously published transcriptomic dataset (Narsai et al., 2017), we found that the majority (*N* = 9; 64%) of *lncCOBRA* transcripts displayed germination-dependent patterns of abundance, with peaks in abundance at various points during seed germination (Supplementary Figure 1B). Going forward, we focused on *lncCOBRA1*, *lncCOBRA3*, and *lncCOBRA5* due to their highly specific abundance profiles during seed germination and the availability of insertional mutant lines for these loci. *lncCOBRA1* and *lncCOBRA3* were most abundant after 48 h of stratification at 4°C in the dark followed by 1 h in light, while *lncCOBRA5* abundance was highest slightly later, with a peak in abundance 6 h after transfer into light conditions (Figure 1A and Supplementary Figure 1B). Abundance of the three *lncCOBRA* transcripts decreased rapidly as the seeds progressed through germination and transitioned into seedlings (Figure 1A and Supplementary Figure 1B). Supporting this, the Arabidopsis expression atlas in the eFP Browser (Klepikova et al., 2016) revealed that all three *lncCOBRA* transcripts were expressed early during seed germination, with the highest expression at 1 day after imbibition (Supplementary Figure 1C). The abundance of *lncCOBRA1*, *lncCOBRA3*, and *lncCOBRA5* was also dynamic throughout seedling development as measured by quantitative reverse transcription-PCR (qPCR), as they had the highest abundance in 2-day-old seedlings and rapidly decreased in abundance as the seedlings aged (Figure 1B and Supplementary Figure 1D).

Additionally, *lncCOBRA* transcripts displayed tissue specific patterns of accumulation. For instance, we found that *lncCOBRA5* abundance is highest in leaf tissue and increases in abundance as the age of the leaf progressed from embryonic cotyledons to juvenile leaves and adult leaves, while *lncCOBRA3* demonstrated similar levels of abundance in all tissues profiled (Figure 1C). In contrast, *lncCOBRA1* had the highest abundance in 5-day-old seedlings, specifically in the cotyledons, and decreased as the leaves increased in age, with a significant (*p*-value < 0.001; Wilcoxon *t*-test) decrease in abundance between 5-day-old cotyledons and true leaves (both juvenile and adult leaves) (Figure 1C and Supplementary Figure 1E). Thus, all three *lncCOBRA* transcripts examined were highly abundant early in germination and decreased as development progressed. In particular, *lncCOBRA1* was highly abundant in embryonic cotyledons and decreased in abundance as true leaves emerge, suggesting *lncCOBRA1* may function during germination and/or early in plant development.

Since these lincRNAs were originally identified as nuclear lincRNAs, and lincRNA function is influenced by subcellular localization, we sought to determine if they were solely nuclear retained. To do so, we isolated pure nuclear and cytoplasmic fractions using the isolation of nuclei tagged in specific cell types (INTACT) technique (Deal and Henikoff, 2010, 2011) and performed qPCR for *lncCOBRA1*, *lncCOBRA3*, and *lncCOBRA5* as well as nuclear (*U6*) and cytoplasmic (*5.8S rRNA* and *18S rRNA*) positive controls. All three *lncCOBRA* transcripts were significantly (*p*-value < 0.001; Wilcoxon *t*-test) enriched in the nuclear fraction like *U6* but not in the cytoplasmic fraction where the two rRNAs were enriched, confirming these transcripts were indeed primarily nuclear localized (Figure 1D).

### *lncCOBRA1* Contains Two Highly Conserved snoRNA Domains and Is Processed at Its 3′ End After Transcription

As both *lncCOBRA1* and *lncCOBRA3* contain small nucleolar RNA (snoRNA) sequences annotated within their transcripts (Figure 1E and Supplementary Figure 1F), and given the evident importance of sno-lncRNAs in humans (Xing and Chen, 2018), we were particularly interested in these two transcripts. Since *lncCOBRA3* lacked tissue-specific patterns of abundance and *lncCOBRA1* had distinct patterns of abundance during seed germination and development, we decided to focus on *lncCOBRA1*. *lncCOBRA1* was annotated to be a 318 nt lincRNA in the Araport11 genome annotation and contained two snoRNA sequence domains within it. The two annotated snoRNA domains overlapped with two previously identified RBP binding sites (Figure 1E; Gosai et al., 2015). These RNA binding/snoRNA domains displayed the highest level of sequence similarity in a sequence alignment of *lncCOBRA1* homologs from five Brassicaceae with *AT1G05917* (sno-*COBRA1A)* and *AT1G05907* (sno-*COBRA1B)* having ∼79 and 56% sequence identity among the profiled species, respectively (Figure 1E). *lncCOBRA1* was highly conserved in all species profiled, with 30–46% sequence identity in the 500 nt up- and downstream of the 5′ most snoRNA (*AT1G05917*), which also included *sno-COBRA1B* (Figure 1E and Supplementary Figure 2A; Geneious | Bioinformatics Solutions for the Analysis of Molecular Sequence Data, 2019). To ensure we are examining the *lncCOBRA1* lincRNA rather than a functional set of snoRNAs, two primer sets were used for all qPCR analyses, one set within sno-*COBRA1A* and the other set (set 2) amplifying the region between the two snoRNAs (Supplementary Figures 1D,E,G; blue and red primers). In addition to their sequence conservation within Brassicaceae, sno-*COBRA1A* and sno-*COBRA1B* have sequence homology to human SNORD59A and SNORD59B, with sequence identity of 76 and 90%, respectively (Liang-Hu et al., 2001; Supplementary Figure 2B). In fact, their tandem orientation is also conserved in humans, with SNORD59A upstream of SNORD59B in an intron of the protein-coding transcript encoding ATP synthase subunit d (ATP5PD) (Kiss-László et al., 1996). Overall, these findings indicate that these snoRNA sequences and orientation are highly conserved, suggesting they are of significant evolutionary importance.

**FIGURE 2:**
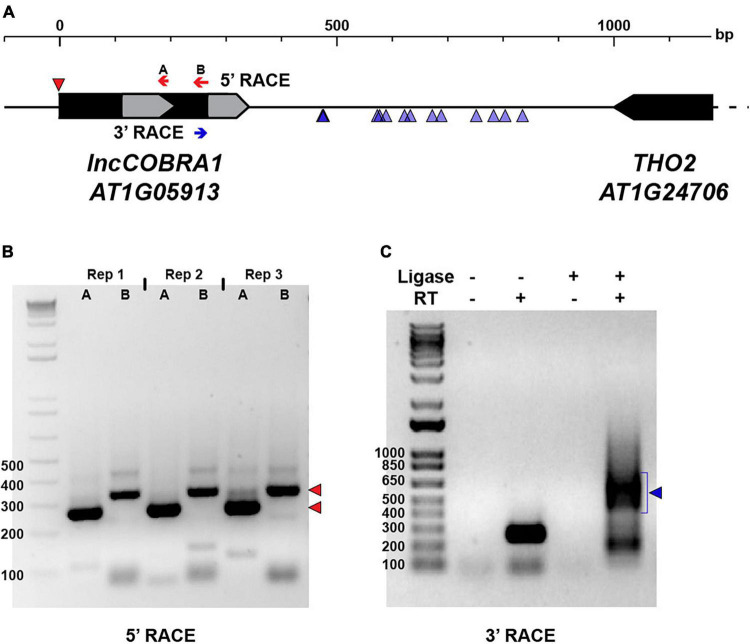
Post-transcriptional processing of *lncCOBRA1.* **(A)** Diagram of *lncCOBRA1* (*AT1G05913*) locus. Gray arrows represent the two snoRNAs annotated within *lncCOBRA1.* Red arrows represent the two primers used for 5′ RACE and red triangle represents the 5′ end identified by 5′ RACE PCR in **(B)**. Blue arrow represents the primer used for 3′ RACE. Blue triangles represent the 3’ most end identified through Sanger sequencing 14 colonies. **(B)** Three biological replicates of 5′ RACE with primers indicated in **(B)**. Red triangles represent the two major bands of PCR product. Ladder is 1 kb + . **(C)** PCR results from 3′ RACE in Col-0 5-day-old seedlings. –/+T4 RNA ligase, –/+SuperScript II. Ladder is 1 kb +.

In humans, sno-lncRNAs are derived from introns excised from protein-coding mRNAs that contain two snoRNA sequences (Xing and Chen, 2018). Instead of being degraded like normal, these introns are debranched and trimmed at the 5′ and 3′ ends by exonucleases until the enzyme reaches the snoRNA domain. The highly structured and protein-bound nature of the snoRNA sequences acts as protection from further degradation, resulting in lncRNAs flanked by snoRNA sequences at each end, but that lack 5′ caps and poly(A) tails (Xing and Chen, 2018). To determine if a similar mechanism was used during *lncCOBRA1* biogenesis, we first performed 5′ rapid amplification of cDNA ends (5′ RACE) to determine the 5′ end of the transcript. In 5′ RACE, any present 5′ caps are removed, and an adapter is directly ligated to the 5′ end of RNA. Following reverse transcription with a gene specific primer and two rounds of PCR, the precise 5′ end of the transcript can be determined (Figure 2A). If the 5′ end of *lncCOBRA1* was as annotated in Araport 11, we would expect PCR products of 250 and 319 bp produced with a primer within the 5′ adapter and two reverse primers, A and B, respectively (Figures 2A,B). Indeed, the 5′ RACE PCR reactions produced products as expected, indicating that the annotated 5′ end of *lncCOBRA1* is indeed where the transcript begins (Figures 2A,B; 5′ RACE results indicated by red triangle), and thus *lncCOBRA1* is apparently not trimmed at the 5′ end after transcription.

We next asked if there was 3′ end processing and sought to determine the full length of *lncCOBRA1.* To begin, we performed RT-PCR with a forward primer at the 5′ most end of the transcribed RNA as confirmed by 5′ RACE and five tiled reverse primers (Supplementary Figure 3A, green arrows). This revealed that *lncCOBRA1* was substantially longer than originally annotated, with amplification of *lncCOBRA1* with all reverse primers, indicating that *lncCOBRA1* is transcribed as a much longer transcript, possibly over 1000 nt long (Supplementary Figure 3B). Given the tissue specificity of *lncCOBRA1* abundance (Figure 1C), we performed the RT-PCR in 2-, 3-, 4-, and 5-day-old seedlings as well as seeds 1- and 2-days-after-imbibition to determine if there were different isoforms in a developmental manner. This revealed amplification with all reverse primers in all developmental time points, revealing that *lncCOBRA1* was over 1000 nt at these stages as well (Supplementary Figure 3B). Overall, this suggests that *lncCOBRA1* is a much longer lincRNA than initially hypothesized.

To determine the precise 3′ end of *lncCOBRA1*, we performed 3′ RACE. Similar to 5′ RACE, an adapter is ligated to the 3′ end followed by reverse transcription with a gene specific primer and two rounds of nested PCR (Figure 2A). The final PCR reaction produced a diffuse band around 500–650 bp in length, which would suggest a 742–892 nt long transcript based on the site of the 3’ RACE primer (Figures 2A, blue arrow, C). Since the resulting 3′ RACE PCR band was diffuse, we extracted the PCR product, cloned it into a sequencing vector and performed Sanger sequencing to identify the precise 3′ end of *lncCOBRA1*. After sequencing 14 independent colonies, several 3′ ends of *lncCOBRA1* were revealed, with the majority of 3′ ends centering ∼250 and ∼350 nt downstream of the 3′ RACE primer (Figure 2A; blue triangles). The various 3′ ends detected by 3′ RACE, the diffuse 3′ RACE PCR band (Figure 2C), and the RT-PCR results (Supplementary Figure 3B) indicate that *lncCOBRA1* is transcribed as a longer transcript, possibly over 1000 nt in length (Supplementary Figure 3B), and is trimmed from its 3′ end to reach a final transcript ∼500–600 nt long, possibly with several stable 3′ ends. Importantly, in all of the 14 colonies sequenced, no polyA tail was identified. This, along with our inability to detect *lncCOBRA1* in any published polyA-selected RNA-seq datasets (data not shown) suggests that *lncCOBRA1* is not polyadenylated in its final processed form.

In plants, polycistronic snoRNAs are encoded in intergenic regions, transcribed by RNA Pol II and generally contain two conserved promoter elements, a *Telo*- box and a Site II element (combined referred to as *Telo*SII) (Gaspin et al., 2010). Notably, in Arabidopsis nearly all ribosomal protein genes and other genes involved in ribosome biogenesis and translation contain *Telo*SII elements in their promoters (Gaspin et al., 2010). This combined *Telo*SII element is found upstream of the TATA box and acts to coordinate expression of snoRNAs and protein-coding genes implicated in ribosome biogenesis (Qu et al., 2015). Interestingly, the *lncCOBRA1* promoter contained both a *Telo*-box and two Site II elements upstream of a TATA-box in the *lncCOBRA1* promoter, suggesting it is regulated in a similar manner to canonical snoRNAs and may be coordinated with genes related to ribosome biogenesis (Supplementary Figure 3C). In addition, the promoter contained a conserved non-coding sequence (CNS) (Velde et al., 2014), which are shown to be highly associated with genes encoding transcription factors and developmental genes and are enriched for transcription factor binding sites (Burgess and Freeling, 2014). The presence of a CNS further emphasizes the conservation of the *lncCOBRA1* gene locus (Supplementary Figure 3C). Overall, *lncCOBRA1* is a highly conserved lincRNA that is trimmed at its 3′ end post-transcriptionally to generate a ∼500–600 nt lincRNA.

### Loss of *lncCOBRA1* Results in Delayed Germination and Smaller Plants

**FIGURE 3:**
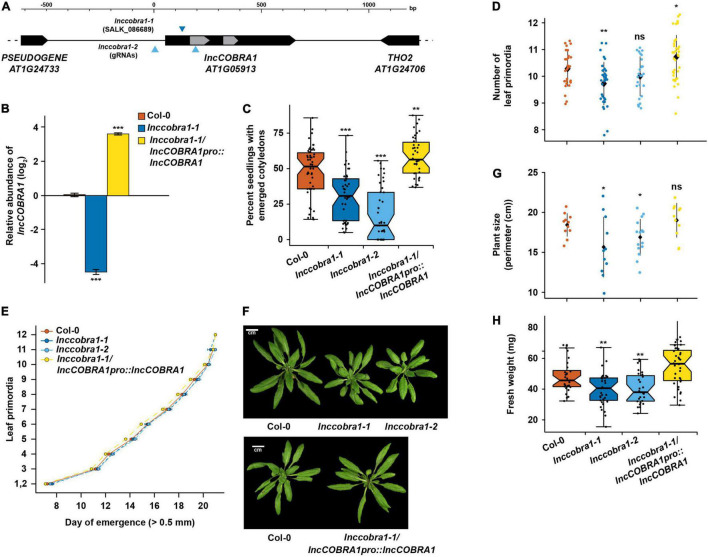
Loss of *lncCOBRA1* results in delayed germination and smaller plants. **(A)** Diagram of *lncCOBRA1* (*AT1G05913*) locus. Gray arrows represent the two snoRNAs annotated within *lncCOBRA1*. Triangles represent the location of the T-DNA insertion in SALK_086689 and location of the two guide RNAs used to generate a CRISPR deletion. **(B)** Relative abundance of *lncCOBRA1* in Col-0, *lnccobra1-1*, and *lnccobra1-1/lncCOBRA1pro:lncCOBRA1*. Abundance is normalized by the geomean of *UBC9* and *UBC10* and relative to Col-0. *** Denotes *p*-value < 0.001; Wilcoxon *t*-test. *N* = 3. Error bars represent SEM. **(C)** Percent of seeds germinated 48 h after sowing. Over 600 seedlings were measured per genotype on over 37 independent plates. *** Denotes *p*-value < 0.001; Wilcoxon *t*-test. **(D)** Number of leaf primordia > 0.5 mm in 3-week-old plants. *N* > 27 plants per genotype. ns, *, and ** denotes *p*-value > 0.05, <0.05, and < 0.01, respectively; Wilcoxon *t*-test. Black diamond represents the mean ± SD. **(E)** Leaf initiation rate. The date was recorded for the first day each leaf primordia was visible by eye, ∼0.5 mm. *N* > 27 plants per genotype. Error bars represent SEM. **(F)** Representative images of 5-week-old Col-0, *lnccobra1-1*, *lnccobra1-2*, and *lnccobra1-1/lncCOBRA1pro:lncCOBRA1*. Plants were grown in a 16/8 h light/dark photoperiod at 22°C. All photos were taken the same day. **(G)** Plant perimeter analysis using ImageJ (see section “Materials and Methods”). *N* > 11 per genotype. * Denotes *p*-value < 0.05; Wilcoxon *t*-test. Black diamond represents the mean ± SD. **(H)** Fresh weight of aerial tissue from 3-week-old plants. *N* > 27 plants per genotype. * and ** denote *p*-value < 0.05 and <0.01, respectively; Wilcoxon *t*-test.

To examine the function of *lncCOBRA1*, we obtained a T-DNA insertion line (*lnccobra1-1;* SALK_086689) from the Arabidopsis Biological Resource Center with an insertion upstream of sno-*COBRA1A* and generated a complete *lncCOBRA1* null (*lnccobra1-2*) using CRISPR gene editing (Figure 3A). PCR and Sanger sequencing confirmed that the CRISPR guide RNAs caused a large deletion of 1325 bp (Supplementary Figures 4A,B). This larger than expected deletion was likely a product of double strand break repair (Korablev et al., 2020) and importantly did not disrupt the surrounding genes.

*lnccobra1-1* had significantly (*p*-value < 0.001; Wilcoxon *t*-test) depleted levels of *lncCOBRA1* as measured by qPCR and *lnccobra1-2* levels were unmeasurable as it is a null mutant with the entire gene deleted (Figure 3B and Supplementary Figure 4C) while levels and processing of rRNAs were minimally affected (Supplementary Figures 4D,E). Furthermore, the T-DNA insertion and CRISPR deletion were specific for decreasing *lncCOBRA1* as levels of the downstream protein-coding gene *THO2* were mostly unaffected in either mutant line (Supplementary Figure 4D). We did identify a slight but significant increase in *5.8S rRNA*, *18S rRNA*, and *25S rRNA* levels, but no visible changes in rRNA processing in the mutants compared to Col-0 (Supplementary Figure 4E). Thus, *lncCOBRA1* likely does not influence rRNA processing even though it contains two well-conserved snoRNA domains.

We also complemented the *lnccobra1-1* background by introducing the entire genomic region between the two neighboring genes into this genetic background (*lncCOBRA1pro:lncCOBRA1*/*lnccobra1-1*; hereafter *lncCOBRA1/lnccobra1-1*). *lncCOBRA1* complementation resulted in a significant increase in *lncCOBRA1* levels (Figure 3B and Supplementary Figure 4C). This overexpression of *lncCOBRA1* eliminated the slight but significant increase in *5.8S rRNA*, *18SrRNA*, *and 25S rRNA* levels observed in the mutant alleles (*p*-value > 0.05; Wilcoxon *t*-test), suggesting that the slight increase in abundance of these mature rRNAs may in fact be due to the loss of *lncCOBRA1* (Supplementary Figure 4D). In total, our findings indicate that both *lnccobra1* mutant lines specifically and significantly decrease the levels of this lincRNA.

Given the high abundance of *lncCOBRA1* during seed germination (Figure 1), we examined the number of seeding with fully emerged cotyledons after 2 days in the growth chamber of Col-0, *lnccobra1-1*, *lnccobra1-2*, and *lncCOBRA1/lnccobra1-1* seeds 48 h after sowing as a proxy for germination defects. We observed that significantly (*p*-value < 0.001; Wilcoxon *t*-test) fewer *lnccobra1-1* and *lnccobra1-2* seeds germinated than in the Col-0 background, while significantly (*p*-value < 0.01; Wilcoxon *t*-test) more *lncCOBRA1/lnccobra1-1* seeds germinated at 48 h (Figure 3C), suggesting that *lncCOBRA1* levels affect seed germination.

The effects of *lncCOBRA1* on germination persisted throughout vegetative growth, as 3-week-old *lnccobra1-1* plants were slightly but significantly (∼0.5 leaves; *p*-value < 0.01; Wilcoxon *t*-test) delayed in leaf production compared to same aged Col-0 plants. This same trend was also observed in *lnccobra1-2* plants, but not to a level of statistical significance (*p*-value > 0.05; Wilcoxon *t*-test) (Figure 3D). Increased levels of *lncCOBRA1* in *lncCOBRA1/lnccobra1-1* plants led to more leaves than Col-0 (∼0.5 leaves, *p*-value < 0.05; Wilcoxon *t*-test) (Figure 3D), suggesting *lncCOBRA1* is responsible for this phenotype. This change in number of leaves at 3-weeks after planting was not due to a change in the overall growth rate of the plants, as there is no change in rate of leaf initiation in *lnccobra1-1*, *lnccobra1-2*, or *lncCOBRA1/lnccobra1-1* compared to Col-0 (Figure 3E). *lnccobra1-1* and *lnccobra1-2* plants were also substantially smaller than Col-0 plants, while the plants overexpressing *lncCOBRA1* (*lncCOBRA1/lnccobra1-1*) rescued this phenotype and resulted in plants that were slightly larger in both 3- and 5-week-old plants (Figures 3F–H and Supplementary Figure 5A). Aside from overall size of the plants, the individual rosette leaves were also smaller in the mutant plant lines (Supplementary Figure 5B). Since altered *lncCOBRA1* levels did not affect the rate of growth (Figure 3E), it is possible that the smaller nature of *lnccobra1-1* and *lnccobra1-2* may be due to a change in either the number or size of leaf cells, though this needs to be probed further. Overall, levels of *lncCOBRA1* effect seed germination, and these germination effects persist through vegetative growth, resulting in plants that are smaller or larger than Col-0 when *lncCOBRA1* levels are decreased or increased, respectively.

### *lncCOBRA1* Interacts With a Wide Variety of Proteins

**FIGURE 4:**
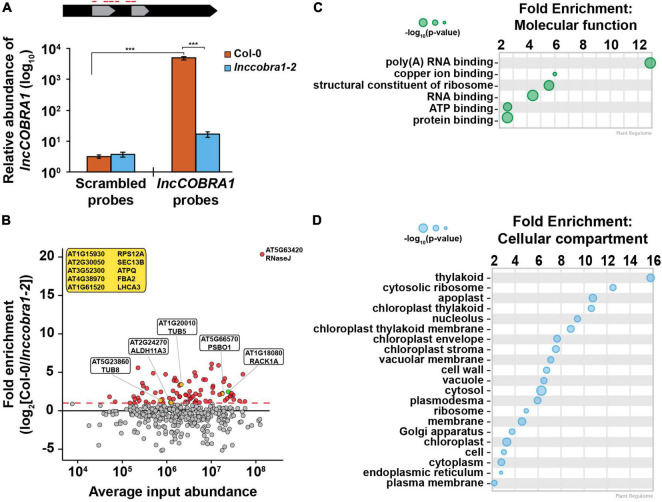
ChIRP enriches for *lncCOBRA1* and identifies 113 *lncCOBRA1*-interacting proteins. **(A)** Relative abundance of *lncCOBRA1* in ChIRP-MS experiments. Abundance normalized by *U6* and is relative to Col-0 input. Error bars represent SEM. ns, *, **, and *** denotes *p*-value > 0.05, < 0.05, < 0.01, and < 0.001, respectively; Wilcoxon *t*-test. *N* = 3. **(B)** Proteins identified from ChIRP followed by MS. *X*-axis is the average protein abundance in Col-0 and *cobra1-2* inputs. *Y*-axis is fold enrichment in Col-0 relative to *lnccobra1-2* with *lncCOBRA1* probes. All dots were enriched with *lncCOBRA1* probes compared to scrambled sequence probes. Red dots indicate proteins enriched with *lncCOBRA1* probes over scrambled and enriched at least 1-fold change in Col-0 compared to *lnccobra1-2*. Green dot represents RACK1A. Yellow dots represent *lncCOBRA1*-interacting proteins that were experimentally shown to interact with RACK1A. Yellow box contains *lncCOBRA1*-interacting proteins that were experimentally shown to interact with RACK1A but were not identified in Col-0 with scrambled probes. *N* = 3. **(C,D)** Gene ontology enrichment analysis for molecular function **(C)** and cellular compartment **(D)** using Plant Regulomics (Ran et al., 2020) for *lncCOBRA1*-interacting proteins. Size of circles represents –log_10_ (*p*-value).

To begin to understand the molecular function of *lncCOBRA1*, we set out to identify what proteins bind *lncCOBRA1*, as *lncCOBRA1* was initially identified for having sites of RBP binding (Supplementary Figure 1A) (Gosai et al., 2015). To do so, we performed chromatin isolation by RNA purification followed by mass spectrometry (ChIRP-MS) (Chu et al., 2015). In this technique, we incubated lysates from 5-day-old Col-0 and *lnccobra1-2* seedlings with biotinylated probes antisense to *lncCOBRA1* (Figure 4A) or a scrambled sequence as a negative control. We then used streptavidin coated beads to pull down *lncCOBRA1*, isolated proteins bound and performed mass spectrometry. We confirmed the efficacy of the pulldown by qPCR and found *lncCOBRA1* was significantly (*p*-value < 0.001; Wilcoxon *t*-test) enriched with probes antisense to *lncCOBRA1* compared to the scrambled sequence control probes, indicating that the *lncCOBRA1* probes are highly specific (Figure 4A). Importantly, enrichment of *lncCOBRA1* with the experimental probes was significantly (*p*-value < 0.001; Wilcoxon *t*-test) depleted when ChIRP was performed in *lnccobra1-2* null seedlings (Figure 4A). As *lncCOBRA1* contains two snoRNA domains, we also asked whether *lncCOBRA1* directly interacted with rRNAs and found that *lncCOBRA1* probes did not enrich for *5.8S rRNA*, *18S rRNA*, or *25S rRNA* relative to scrambled sequence control probes (Supplementary Figure 6A). This indicated that *lncCOBRA1* does not interact with rRNA, further confirming that the snoRNA domains within *lncCOBRA1* do not function like canonical snoRNAs (Supplementary Figure 6A).

After mass spectrometry, we set out to identify high confidence interactors from the ∼2200 proteins identified (Supplementary Data Set 1A). To do so, we required that proteins must be (1) identified in at least 2 biological replicates of the *lncCOBRA1* pulldown in Col-0 plants (*N* = 469 proteins) (Supplementary Data Set 1B), (2) enriched with the *lncCOBRA1* probes compared to scrambled sequence control probes (*N* = 206), and (3) enriched > 2-fold in Col-0 compared to *lnccobra1-2* seedlings (*N* = 74; Figure 4B, red dots an Supplementary Figure 6B). A total of 74 proteins were identified from these filtering steps. An additional 39 proteins were identified in at least 2 biological replicates in the *lncCOBRA1* pulldown but absent from control pulldowns (scrambled or *lnccobra1-2* background; Table 2). In total, 113 proteins were identified as high-confidence *lncCOBRA1*-interacting proteins, and specifically bound to *lncCOBRA1* in 5-day-old Col-0 seedlings.

**TABLE 2:** Proteins not identified in pulldown with scrambled probes in Col-0.

Locus	ID
AT5G09660	PMDH2
AT1G59870	PEN3
ATCG00800	RPS3
AT3G52300	ATPQ
ATCG00160	RPS2
AT4G16143	IMPA-2
AT1G54030	MVP1
AT1G06760	HISTONE1.1
AT4G38970	FBA2
AT3G46970	PHS2
AT1G12270	HOP1
AT1G20020	FNR2
AT3G47800	AT3G47800
AT2G32080	PUR ALPHA-1
AT4G38630	RPN10
AT4G27440	PORB
AT3G08740	AT3G08740
AT1G60000	AT1G60000
AT3G42050	AT3G42050
AT1G17470	DRG1
AT5G19350	AT5G19350
AT5G58140	PHOT2
AT1G64550	GCN3; ABCF3
AT3G22640	PAP85
AT3G08530	AT3G08530
AT1G61520	LHCA3
AT3G13580	AT3G13580
AT1G15930	AT1G15930
AT2G30050	AT2G30050
AT4G32840	PFK6
AT1G51060	HTA10
ATCG00820	RPS19
AT1G59900	E1 ALPHA
AT4G36690	ATU2AF65A
AT1G09750	AT1G09750
AT1G49600	RBP47A
AT4G22140	EBS
AT5G05470	EIF2 ALPHA
AT5G36880	ACS

*lncCOBRA1*-interacting proteins were significantly enriched for proteins with molecular function of RNA binding, and 37.5% (*p*-value < 5.21 × 10^–40^; hypergeometric test) were demonstrated to bind to RNA in a recent study identifying the RNA binding proteome of Arabidopsis leaves (Bach-Pages et al., 2020), supporting the claim that these proteins interact directly with *lncCOBRA1* (Figure 4C and Supplementary Figure 6C). Those proteins not demonstrated to have RNA binding capabilities may still interact with *lncCOBRA1* indirectly. In addition, several proteins involved in transcription regulation were identified, including PUR ALPHA-1 (PURα), which has hypothesized roles in rRNA transcription (Table 3; Trémousaygue et al., 2003).

**TABLE 3:** COBRA1-interacting proteins involved in transcriptional regulation.

Locus ID	Description	Common name
AT1G06760	Winged-helix DNA-binding transcription factor family protein	HISTONE 1.1 (H1.1)
AT1G51060	Encodes HTA10, a histone H2A protein	HISTONE H2A 10 (HTA10)
AT1G54060	Member of the trihelix DNA binding protein family. Involved in repressing seed maturation genes during seed germination and seedling development.	6B-INTERACTING PROTEIN 1-LIKE 1 (ASIL1)
AT1G61730	DNA-binding storekeeper protein-related transcriptional regulator	ABNORMAL SUSPENSOR 2 (SUS2)
AT1G80070	Encodes a factor that influences pre-mRNA splicing and is required for embryonic development. Mutations result in an abnormal suspensor and embryo lethality	
AT2G32080	Similar to the conserved animal nuclear protein PUR alpha which was implicated in the control of gene transcription and DNA replication	PURIN-RICH ALPHA 1 (PUR ALPHA-1)
AT3G46780	Plastid transcriptionally active 16	PLASTID TRANSCRIPTIONALLY ACTIVE 16
AT3G51800	Putative nuclear DNA-binding protein G2p (AtG2) mRNA	ATG2
AT3G61310	AT hook motif DNA-binding family protein	AT-HOOK MOTIF NUCLEAR
AT4G22140	Encodes a chromatin remodeling factor that regulates flowering time.	EARLY BOLTING IN SHORT DAYS (EBS)
AT4G35800	Encodes the unique largest subunit of nuclear DNA-dependent RNA polymerase II; the ortholog of budding yeast RPB1 and a homolog of the E. coli RNA polymerase beta prime subunit.	RNA POLYMERASE II LARGE SUBUNIT (NRPB1)
AT4G36690	Regulates flowering time and displays a redundant role in pollen tube growth together with AtU2AF65b.	ATU2AF65A
AT5G55220	Trigger factor type chaperone family protein	TIG1
AT5G56900	CwfJ-like family protein/zinc finger (CCCH-type) family protein	

*lncCOBRA1*-interacting proteins were involved in a wide-range of biological functions, including response to cytokinin and abscisic acid (ABA), gluconeogenesis, and photorespiration (Supplementary Figure 6D; Ran et al., 2020). Additionally, *lncCOBRA1-*interacting proteins were enriched for proteins functioning in “structural constituents of the ribosome” and located in the cytoplasmic ribosome, chloroplasts, and the nucleolus (Figure 4D). In fact, twelve of the *lncCOBRA1-*interacting proteins (10.6%; *p*-value < 2.7 × 10^–13^; hypergeometric test; Table 4) were identified in a previous study identifying the nucleolar proteome (Pendle et al., 2005). The nucleolus is a non-membrane bound nuclear structure that is the site for ribosome assembly and maturation. Given the snoRNA domains in *lncCOBRA1* and the identification of cytoplasmic ribosomal constituents bound to the nuclear localized *lncCOBRA1*, we hypothesize that *lncCOBRA1* may be localized to the nucleolus. Among these RNA binding *lncCOBRA1*-interacting proteins is RNaseJ, which is the most enriched protein bound to *lncCOBRA1* in Col-0 relative to *lnccobra1-2* (Figure 4B). RNaseJ is a metallo-beta-lactamase protein that possesses endo- and 5′-3′ exonuclease activities in bacteria and chloroplasts within plants and is required for embryo and chloroplast development (Halpert et al., 2019) with roles in rRNA maturation and 5′ stability of mRNAs in bacteria (Mathy et al., 2007). This finding provides an additional connection between *lncCOBRA1* and ribosome processing.

**TABLE 4:** Nucleolar lncCOBRA1-interacting proteins.

Locus	ID	Description
AT1G61730	AT1G61730	DNA-binding storekeeper protein-related transcriptional regulator
AT2G30050	AT2G30050	Transducin family protein/WD-40 repeat family protein
AT3G51800	ATG2	ERBB-3 BINDING PROTEIN 1 (EBP1); (ATG2)
AT5G42020	BIP2	(BIP2);LUMINAL BINDING PROTEIN (BIP)
AT3G52300	ATPQ	ATP SYNTHASE D CHAIN, MITOCHONDRIAL (ATPQ)
AT1G54060	ASIL1	6B-INTERACTING PROTEIN 1-LIKE 1 (ASIL1)
AT3G08580	AAC1	ADP/ATP CARRIER 1 (AAC1)
AT1G02780	EMB2386	EMBRYO DEFECTIVE 2386 (emb2386); Ribosomal protein L19e family protein
AT1G51060	HTA10	HISTONE H2A 10 (HTA10)
AT4G16143	IMPA-2	IMPORTIN ALPHA ISOFORM 2 (IMPA-2)
AT2G33150	KAT2;	POTASSIUM CHANNEL IN ARABIDOPSIS THALIANA 2 (KAT2)
	PED1	PEROXISOME DEFECTIVE 1 (PED1)
	PKT3	PEROXISOMAL 3-KETOACYL-COA THIOLASE 3 (PKT3)
AT3G53020	STV1; RPL24B	SHORT VALVE1 (STV1);RIBOSOMAL PROTEIN L24 (RPL24B)

### *lncCOBRA1*-Interacting Proteins Are Highly Interconnected

As proteins tend to act in complexes and *lncCOBRA1*-interacting proteins were enriched for proteins involved in protein binding (Figure 4C), we next asked if there were known interactions among the 113 *lncCOBRA1*-interacting proteins (Figure 4B and Table 2). Using STRING, we generated a protein–protein interaction (PPI) network which formed significantly (*p*-value < 1.0 × 10^–16^; STRING) more interactions than expected, indicating that *lncCOBRA1*-interacting proteins had more interactions among themselves than what would be expected for a random set of proteins of a similar size from the Arabidopsis proteome (Supplementary Figure 7A; Szklarczyk et al., 2019). Using k-means clustering, the proteins within the network were further grouped into 5 clusters (green, cyan, blue, red, and yellow) (Supplementary Figures 7A,B). Each cluster represented distinct groups of proteins with cytokinin response-related and photosynthetic proteins, glycolytic proteins, and mRNA splicing-related proteins clustering together to form the green, cyan and blue clusters, respectively (Huang et al., 2009). Of the five clusters, blue, green, and cyan were interlaced throughout the network, and hard to distinguish between each other. The red cluster was the most spread out, lying on the periphery of the network with very little significant enrichment for biological processes or cellular compartments, indicating this cluster represents a variety of different proteins with a range of functions (Supplementary Figure 7B).

Within the red cluster lies the trihelix DNA binding transcription factor 6B-INTERACTING PROTEIN 1-LIKE (ASIL1) (Supplementary Figure 7A), which was previously shown to be involved in repressing seed maturation genes during seed germination and seedling development (Gao et al., 2009) and was also previously identified in the nucleolus (Table 2). Since numerous nuclear lincRNAs function in gene regulation by binding and directing transcription factors to the correct genomic loci, and ASIL1 regulates germination, which is mis-regulated in *lnccobra1-1* and *lnccobra1-2* plants, it is possible that *lncCOBRA1* interacts with ASIL1 to affect seed maturation genes during seed germination and seedling development but further studies are required to test this.

**FIGURE 5:**
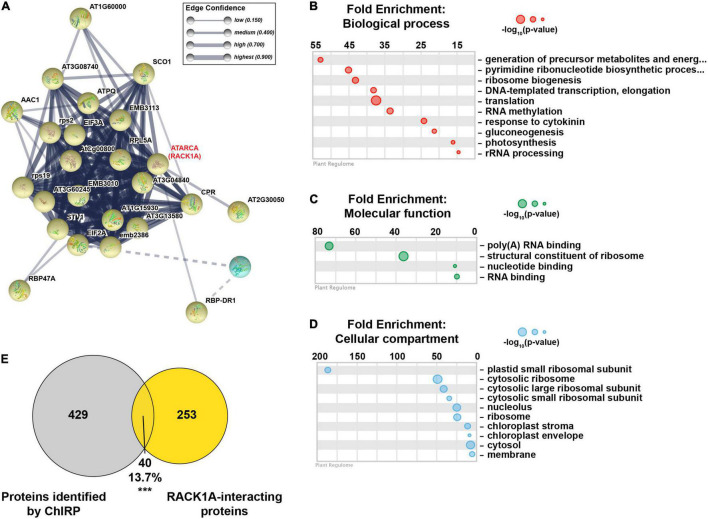
*lncCOBRA1* interacts with RACK1A and a tight network of proteins related to ribosome biogenesis. **(A)** Yellow protein-protein interaction k-means cluster generated from STRING (Szklarczyk et al., 2019). Thickness of lines connecting notes indicates the confidence of that protein–protein interaction. Dotted line indicates interaction with a different cluster (see Supplementary Figure 7 for full network). **(B–D)** Gene ontology enrichment analysis for biological process **(B)**, molecular function **(C)**, and cellular compartment **(D)** using Plant Regulomics (Ran et al., 2020) for *lncCOBRA1*-interacting proteins in the yellow cluster. Size of circles represents –log_10_ (*p*-value). **(E)** Overlap between proteins identified in at least two biological replicates of Col-0 ChIRP with the *lncCOBRA1* probes and proteins identified as RACK1A binding. *** denotes *p*-value < 0.001; Hypergeometric test.

A closer examination of the yellow cluster, which was the most compact group (Figure 5A), revealed that this close network was enriched for proteins involved in ribosome biogenesis, rRNA processing, response to cytokinin, RNA binding, and constituents of the ribosome (Figures 5B–D and Supplementary Figure 7B). This cluster was also enriched for proteins localized in the nucleolus and ribosome (Figures 5B–D and Supplementary Figure 7B). A major node within the yellow cluster was RECEPTOR FOR ACTIVATED C KINASE 1A (RACK1A; encoded by *ATARCA*) (Figure 5A). RACK1A is a major subunit of RACK1, which is a highly conserved scaffold protein present in all eukaryotic organisms studied, from *Chlamydomonas* to plants and humans (Adams et al., 2011). Several proteomics studies have identified a total of 293 proteins that interact with RACK1A (Stark et al., 2006; Olejnik et al., 2011; Kundu et al., 2013; Speth et al., 2013; Cheng et al., 2015; Guo et al., 2019), 40 of which (13.7%; *p*-value < 2.1 × 10^–28^; hypergeometric test) were identified in at least 2 biological replicates of *lncCOBRA1* pulldown in Col-0 (Figure 5E). This included RACK1B, another major subunit of RACK1 (Guo and Chen, 2008). Nearly 25% of the identified RACK1A-interacting proteins that were identified in ChIRP were specifically bound to *lncCOBRA1* in Col-0 compared to *lnccobra1-2* (*N* = 9; Figure 4B, yellow dots), providing strong evidence that *lncCOBRA1* interacts with RACK1A.

## Discussion

In this study, we use genetic, biochemical, and proteomic analyses to describe a highly conserved, previously uncharacterized sno-lincRNA with functions in seed germination and development. We reveal that *lncCOBRA1* is a ∼500–600 nt lincRNA with germination-, developmental-, and tissue-specific patterns of abundance, with high abundance early during seed germination and decreases as development progresses. Further, we demonstrate that loss of *lncCOBRA1* results in delayed cotyledon emergence and overall smaller plants. We demonstrate that *lncCOBRA1* interacts with a wide variety of proteins, including many nucleolar proteins and scaffold proteins, including the highly conserved RACK1 subunit RACK1A, leading to an overall hypothesis that *lncCOBRA1* acts as a scaffold to bring together proteins involved in several different processes to ultimately regulate plant germination and development.

### Identification of Highly Conserved, Protein-Bound Nuclear lincRNAs From Transcriptome-Wide Analyses

Here, we describe a set of lincRNAs named *CONSERVED IN* BRASSICA RAPA *1-14* (*lncCOBRA1-14*) that were identified for their interactions with nuclear RBPs and sequence conservation in *Brassica rapa* (Supplementary Figure 1A; Gosai et al., 2015). Of the 14 *lncCOBRA* transcripts profiled, 9 contained one or more snoRNAs annotated within it, revealing a previously unidentified class of lincRNAs containing snoRNAs (sno-lincRNAs) in Arabidopsis (Table 1). snoRNAs are a family of conserved nuclear small RNAs (70–200 nt) that are usually concentrated in the Cajal bodies or nucleolus. They traditionally function to modify rRNA or participate in the processing and maturation of ribosomal subunits, where binding of core nucleolar proteins protects the mature snoRNAs and aids in proper function (Rodor et al., 2010). Despite having two snoRNA domains, we do not observe any function of *lncCOBRA1* in rRNA processing (Supplementary Figure 4E), similar to mammalian sno-lncRNAs described previously (Yin et al., 2012).

We predict that the presence of snoRNA sequences in these lincRNAs likely results in their interaction with RBPs, as the annotated snoRNA domains overlap with the protein-bound sites identified previously, and snoRNA sequences are known to be highly protein-bound. Additionally, since snoRNAs are nuclear retained (Figure 1), we predict that the snoRNA sequences contained in these *lncCOBRA* transcripts permit their nuclear retention, though future experiments are needed to test this hypothesis. Most *lncCOBRA* transcripts demonstrated specific patterns of abundance during seed germination. Interestingly, *lncCOBRA* lincRNAs that lacked snoRNA sequences demonstrated the least specificity in abundance patterns during germination (*lncCOBRA8, 9, 13*, and *14*) (Supplementary Figure 1B). Ultimately, this suggests that sno-lincRNAs may be important for germination in Arabidopsis, while conserved, protein-bound lincRNAs that lack snoRNAs may function in different biological processes.

In mammals, the majority of functional snoRNAs are encoded within introns and processed from excised and debranched introns by exonucleolytic trimming. Similarly, all identified mammalian sno-lncRNAs are generated from excised introns as well (Xing and Chen, 2018). In Arabidopsis, while identified snoRNAs in Arabidopsis appear to be homologs of yeast and animal counterparts, they are not encoded within introns but are instead primarily transcribed from intergenic regions as polycistronic gene clusters. As such, the *lncCOBRA* sno-lincRNAs described here are also transcribed from intergenic regions throughout the genome. Thus, *lncCOBRA* sno-lincRNAs represent a previously uncharacterized class of lincRNAs with potentially important biological functions that warrant future studies.

### Regulation of *lncCOBRA1* Transcription

*lncCOBRA1* contains several conserved elements within its promoter known to be present in the promoters of genes involved in ribosome biogenesis and translation. This includes Telo-box and Site II elements (*Telo*SII) (Supplementary Figure 3C). Interestingly, the Telo-box is known to be bound by the *lncCOBRA1*-interacting transcription factor PUR ALPHA-1 (PURα) (Tables 2, 3; Tremousaygue et al., 1999). PURα is a homolog of the animal nuclear protein PUR ALPHA (*PURA*) which is a member of the sequence-specific single-stranded nucleic acid-binding Pur family of proteins. The amino acid sequence of Pura is extraordinarily conserved in sequence from bacteria through humans, where it functions as a transcriptional activator, and as an RNA transport protein. While less is known about PURα in Arabidopsis, it was identified to be an RBP (Bach-Pages et al., 2020) and was previously demonstrated to interact with TEOSINTE BRANCHED 1, CYCLOIDEA, PCF (TCP)-DOMAIN FAMILY PROTEIN 20 (TCP20) (Trémousaygue et al., 2003). TCP20 also binds *Telo*SII elements and regulates expression of ribosomal protein genes (Trémousaygue et al., 2003). In Arabidopsis, nearly all ribosomal protein genes and other genes involved in ribosome biogenesis and translation contain *Telo*SII elements in their promoters (Gaspin et al., 2010). This combined *Telo*SII element is found upstream of the TATA box and acts to coordinate expression of snoRNAs and ribosome biogenesis (Qu et al., 2015). Thus, the interaction between PURα and *lncCOBRA1* could suggest the *lncCOBRA1* binds to PURα to regulate its own expression. Additionally, the presence of the *Telo*SII elements in the *lncCOBRA1* promoter suggests that *lncCOBRA1* may be expressed in a coordinated manner with ribosomal proteins, implicating it in ribosome biogenesis.

### *lncCOBRA1*-Interacting Proteins May Mediate Germination Phenotype Observed in Mutants

RACK1 is a versatile scaffold protein that can bind to numerous signaling molecules from diverse signal transduction pathways (Guo et al., 2007). In Arabidopsis, RACK1 plays an important role in maintaining 60S ribosome biogenesis and 80S monosome assembly, as *rack1a rack1b* double mutants have a decrease in abundance of the 60S ribosomal subunit and 80S monosomes, but no differences in polysomes, suggesting a role for RACK1 in ribosome biogenesis (Guo et al., 2011). Since RACK1A interacts with ribosomal proteins, generally affects translation and responds to several hormones, this suggests that RACK1 has a dual role in signaling and translation, as observed previously for the RACK1 homolog in mammals (Guo et al., 2011).

Additionally, mutants in RACK1A had smaller rosette leaf size and delayed flowering and leaf development under short day conditions (8/16 h photoperiod) (Chen et al., 2006). When grown under long day conditions (16/8 h photoperiod), many of the strong phenotypes observed under short day were alleviated and *rack1a* plants grew at similar rates to wild type, but had slightly smaller rosette leaf size, a phenotype that was exacerbated when additional subunits of RACK1 were deleted (Wang et al., 2019). Overall, *rack1a* plants grown under long day conditions appear to phenocopy *lnccobra1* mutants, suggesting a functional link between RACK1A and *lncCOBRA1*. Moreover, *rack1a* mutants were hypersensitive to ABA (Chen et al., 2006; Guo et al., 2009, 2011) and insensitive to gibberellin (GA) (Chen et al., 2006; Fennell et al., 2012), suggesting a role of RACK1A in regulating seed germination and development. Ultimately, the hypersensitivity of *rack1a* to ABA suggests that RACK1A negatively regulates ABA-mediated seed germination and development.

Given the evidence of RACK1-*lncCOBRA1* interaction (Figures 4, 5) along with similarities in the phenotype of null mutants (Chen et al., 2006; Guo et al., 2019) and protein binding partners (Figures 4, 5), this provides further evidence of a functional link between RACK1A and *lncCOBRA1*, suggesting the possibility that *lncCOBRA1* functions with RACK1A as a scaffold to regulate plant germination and development. Though future studies are required, we propose a hypothesis that *lncCOBRA1* is localized to the nucleolus, where it functions as a scaffold to interact with RACK1A and associated ribosomal proteins to affect ribosome biogenesis. Disruption of *lncCOBRA1* abundance results in disruption of the RACK1 complex and its association with ribosomal proteins, resulting in decreased ribosome biogenesis and the phenotypes observed.

### RNase J Is the Highest Enriched Protein-Bound to *lncCOBRA1*

The protein with the highest enrichment for *lncCOBRA1* binding in Col-0 relative to *lnccobra1-2* was RIBONUCLEASE J (RNASE J; RNJ) (Figure 4B). *RNJ* encodes a metallo-beta-lactamase protein that possesses endo- and 5′-3′ exonuclease activities in bacteria and chloroplasts within plants and is required for embryo and chloroplast development (Halpert et al., 2019). While RNase J plays important roles in rRNA maturation and 5′ stability of mRNAs in bacteria (Mathy et al., 2007), it does not function in the cleavage of polycistronic rRNAs or mRNA precursors in Arabidopsis (Sharwood et al., 2011). Instead, loss of RNase J resulted in a massive accumulation of antisense RNAs, suggesting that RNase J is responsible for degradation of these RNAs generated by the inability of chloroplast RNA polymerase to terminate transcript effectively. The antisense RNAs would otherwise form duplexes with sense strand transcripts and prevent translation (Sharwood et al., 2011). While RNase J is described to be chloroplast localized, it is also predicted to be located in the nucleus by computational predictions (Kaundal et al., 2010). Further, previously, we previously identified a protein thought to be solely chloroplast localized in the nucleus (Gosai et al., 2015). Thus, it is possible that RNase J is in the nucleus, though this needs to be directly experimentally validated.

RNases are essential for non-coding RNA processing and each RNase can have a multitude of targets. For example, RNase P is an endoribonuclease canonically functions to process the 5′ termini of pre-tRNAs but can also cleave other tRNA like structures in the 3′ end of lncRNAs to form mature 3′ ends (Wilusz et al., 2008, 2011; Sunwoo et al., 2009). Additionally, RNase mitochondrial RNA processing (MRP) was originally identified as an RNA-protein endoribonuclease that processes RNA primers of DNA replication in the mitochondria but is actually predominantly found in the nucleolus where it participates in pre-rRNA processing (Lee et al., 1996). Thus, it is possible that RNase J possesses additional functions than previously described, possibly mediated by its interaction with *lncCOBRA1*. Given its function in ribosome maturation in bacteria and the multiple functions of RNases on ncRNAs described previously, we posit that RNase J may have additional function in sno-lincRNA processing in Arabidopsis, specifically the 3′ end processing we observe for *lncCOBRA1*, but future studies will be required to support this hypothesis.

In total, using transcriptome-wide analyses we identified functional candidate lncRNAs based on sequence conservation and the presence of RBP binding sites. We further show the loss of *lncCOBRA1* results in growth phenotypes. While future studies are required, we provide evidence that *lncCOBRA1* interacts with a plethora of proteins involved in many different processes. Overall, we hypothesize that *lncCOBRA1* acts as a scaffold to bring together many different proteins to regulate normal biological processes, including ribosome biogenesis.

## Materials and Methods

### Plant Materials and Growth Conditions

All plants were of the Columbia-0 ecotype and were grown in controlled chambers with a cycle of 16 h light and 8 h dark at 22°C. All seeds used for plate growth were sterilized in 100% ethanol for 1-min followed by a 10-min wash with 30% Clorox and 0.01% Tween-20 solution and rinsed five times with sterilized water. Seeds were then plated and grown on 1/2 MS agar plates with 1% sucrose and 0.8% Phytoblend and stratified by cold treating at 4°C for 48 h then placed in growth chambers with the parameters noted above.

*lncCOBRA1* was previously referred to as *AT1NC031460* in Liu et al. (2012) and *AT1G05913* in the Araport11 genome annotation. *lnccobra1-1* (SALK_086689) was purchased from the Arabidopsis Biological Resource Center and backcrossed once to Col-0, segregated, and homozygous mutants obtained and validated by PCR. RT-qPCR was used to validate significant depletion in the abundance of *lncCOBRA1*.

### CRISPR/Cas9 Plasmid Construction and Mutation Identification

To generate *lnccobra1-2*, the suite of plasmids designed for multiplexed CRISPR genome editing by Lowder et al. (2015) were acquired from Addgene^1^ and used to generate Arabidopsis CRISPR-Cas9 transformation vectors (Lowder et al., 2015). Two different guide RNAs were designed using the CRISPRdirect website^2^ targeting *AT1G05913*. Because Cas9 was chosen to perform genome editing, 5′ -NGG- 3′ was used as the protospacer adjacent motif (PAM) sequence requirement. The *Arabidopsis thaliana* TAIR10 genome was used to ensure the specificity of chosen guide RNAs. The first guide RNA (protospacer sequence: 5′-TATGATTTGATCATCATCGG-3′) is located approximately 50 base pairs upstream of the *AT1G05913* transcription start site, and the second guide RNA (protospacer sequence: 5′-TATATGGCTCTGGAAGAGGG-3′) is located approximately 121 base pairs downstream of the *AT1G05913* transcription start site. Complimentary oligos were designed for each protospacer that contained overhangs compatible with the Arabidopsis *U6* promoter driven guide RNA vectors designed by Lowder et al. (2015) (vectors pYPQ131-pYPQ134) (Lowder et al., 2015).

To generate a CRISPR-Cas9 transformation vector containing two guide RNAs targeting *AT1G05913*, the cloning procedures provided by Lowder et al. (2015) were followed (Lowder et al., 2015). Briefly, each protospacer sequence described above was annealed using complimentary oligos to create a double stranded DNA fragment and then ligated into the vectors pYPQ131 and pYPQ132, respectively. pYPQ131 and pYPQ132 with correctly inserted protospacer sequences were used in a Golden Gate assembly reaction with pYPQ142 to generate a Gateway-compatible entry vector. The pYPQ142 vector with both guide RNAs correctly inserted, along with pYPQ154 carrying an Arabidopsis codon optimized Cas9, and pUBQ10:GW (Stock CD3-1947 from the Arabidopsis Biological Resource Center) were used in a Gateway LR reaction (Thermo Fisher Scientific; Carlsbad, CA, United States) to generate the final transformation vector. The final vector was transformed into wild type *Arabidopsis thaliana* (Col-0) using the floral dip method (Clough and Bent, 1998).

Successful transformants were selected using Glufosinate-ammonium and allowed to set seed to acquire second generation transformants (T2). T2 plants were genotyped to test for a deletion in *AT1G05913* using the PCR primers 5′–CGCTTGTTCAACTCCAAAAAG-3′ and 5′-TTTTGGTATATAAGCTGATGGC-3′. A large band shift was detected in one T2 plant (wild type product size: 1,600 bp, observed product size: approximately 200 bp) (Supplementary Figure 4A), and Sanger sequencing confirmed the deletion to be 1,325 bp. All primers are listed in Supplementary Data Set 2.

### Plasmid Construction and Generation of *lncCOBRA1/lnccobra1-1*

To generate *lncCOBRA1* promoter: *lncCOBRA1/lnccobra1-1*, the entire 1509 bp between the two neighboring genes was amplified from Col-0 genomic DNA and cloned into *Bsp*EI and *Bst*EII restriction enzyme sites of pCAMBIA3301. Transgenic plants were obtained and selected as previously described (Zhang et al., 2006). All primers are listed in Supplementary Data Set 2.

### RNA Extraction

RNA was extracted from the tissues denoted using a liquid nitrogen cooled mortar and pestle. Ground, frozen tissue was transferred to Qiazol lysis reagent (Qiagen; Valencia, CA, United States) and further homogenized using QIAshredders (Qiagen; Valencia, CA, United States). RNA was then isolated using the miRNeasy mini columns as described by the manufacturers’ protocol (Qiagen; Valencia, CA, United States). Following elution from the miRNeasy column, RNA was treated with RNase-free DNase (Qiagen, Valencia, CA, United States) for 25 min at room temperature, ethanol precipitated and resuspended in nuclease-free water supplemented with 1.25% RNaseOUT (Life Technologies; Carlsbad, CA, United States).

### RT-qPCR

All reverse transcription (RT) reactions were performed using SuperScript II following the manufacturers’ instructions with 2.5 mM Random Hexamers (Qiagen; Valencia, CA, United States), 100 units SuperScript II and 30 units RNaseOUT (Invitrogen; Carlsbad, CA, United States) for 2 min at 25°C, 90 min 42°C, 5 min 95°C, hold at 4°C. Before qPCR, cDNA was diluted 1:10 for all RT-qPCR reactions except for ChIRP in which the RT reaction was diluted 1:5.

qPCR was performed with 2X SYBR Green qPCR Master Mix with Rox #2 (Bimake; Houston, TX, United States), as follows per well: 10 μL 2X SYBR Green Master Mix, 1.5 μL cDNA (diluted 1:10), 0.4 μL Rox #2. 2.1 μL water, 6 μL combined 1.5 μM forward and reverse primers. All qPCR reactions were performed in three technical replicates and all primers tested using water to detect background signal and melt curves were analyzed for a single peak. All qPCRs were run using the following program: 95°C for 10 min; 40 cycles of 95°C 30 s, 55°C 30 s, 72°C 30 s. Melt curves were generated by heating the final PCR 1.6°C/s to 95°C for 15 s, decreasing the temperature to 60°C at 1.6°C/s and slowly increasing back to 95°C at 0.1°C/s. Unless otherwise noted, all qPCR experiments were normalized to the geomean of *UBC9* and *UBC10*. All primers are listed in Supplementary Data Set 2.

### Isolation of Nuclei Tagged in Specific Cell Types

To examine RNA abundance in nuclei and cytoplasmic fractions, seeds ubiquitously expressing a biotin ligase receptor peptide fusion protein that is targeted to the nuclear envelope (UBQ10:NTF/ACT2p:BirA Columbia-0 ecotype) were used (Deal and Henikoff, 2010, 2011). After 7 days, seedlings were collected, and flash frozen in liquid nitrogen and stored at –80°C for further processing. The isolation of nuclei tagged in specific cell types (INTACT) (Deal and Henikoff, 2010, 2011) technique was used to isolate pure nuclear and cytoplasmic fractions and RNA extracted before RT and qPCR as described above.

### Tissue Collection

For the germination time course, seedlings were collected 2, 3, 4, 5, 7, and 10 days after stratification and flash frozen in liquid nitrogen and stored in –80°C for further processing. Tissues from 5-week-old Col-0 plants were collected, flash frozen in liquid nitrogen, and stored in –80°C until processing for examining the tissue specificity of *lncCOBRA1* abundance. The sample of adult leaves included a mix of rosette leaves older than leaves 1–4 which were denoted juvenile leaves.

### Brassicaceae *lncCOBRA1* Sequence Alignments

To identify putative sequence homologs of the *AT1G05913* gene, the entire Arabidopsis cDNA sequence was used as query for BLAST using CoGeBlast^3^ using default parameters (*E*-value: 1e-5, Word size: 8, Gap Costs: Existence-5 Extension-2, Match/Mismatch Scores: 1, -2) against representative Brassicaceae species. The top hits for each species were selected based on e-value and quality score and used for subsequent sequence alignments. Selected sequences were aligned using Geneious Prime (Geneious | Bioinformatics Solutions for the Analysis of Molecular Sequence Data, 2019) with the Multiple Alignment tool, utilizing the Geneious Alignment default parameters (Alignment type: Global alignment with free end gaps, Cost Matrix: 70% similarity, Gap open penalty: 12, Gap extension penalty: 3, Refinement iterations: 2).

### Rapid Amplification of cDNA Ends

#### 5′ Rapid Amplification of cDNA Ends

Five μg of RNA from 5-day-old seedlings was first treated with 1 unit of Shrimp Alkaline Phosphatase (SAP; USB Products, Affymetrix, Inc.; Cleveland, OH, United States) in 1X SAP buffer provided and supplemented with 1 mM DTT and 60 units RNaseOUT (Invitrogen, Carlsbad, CA, United States) for 1 h at 37°C. The SAP reaction was inactivated for 15 min at 65°C and the RNA ethanol precipitated overnight. To remove any 5′ m^7^G caps, 500 ng of the SAP-treated RNA was treated with 12.5 units RNA 5’ Pyrophosphohydrolase (RppH; New England BioLabs; Ipswitch, MA, United States) in 1X T4 RNA Ligase Buffer (New England BioLabs; Ipswitch, MA, United States) supplemented with 20 units RNaseOUT (Invitrogen; Carlsbad, CA, United States) in a total reaction volume of 10 μL for 1 h at 37°C and stored at –20°C overnight.

On the following day, the 5′ adapter was added. To the 10 μL RppH reaction, we added 1 μL of 5′ RNA adapter (25 μM; RA5; 5′-GUUCAGAGUUCUACAGUCCGACGAUC-3′) that was first heated to 70°C for 2 min followed by 2 min on ice to relieve secondary structures, 1 μL 10 mM ATP (New England BioLabs; Ipswitch, MA, United States), 10 units T4 RNA Ligase 1 (New England BioLabs; Ipswitch, MA, United States), 1 μL T4 RNA Ligase Buffer (New England BioLabs; Ipswitch, MA, United States), and 40 units RNaseOUT (Invitrogen; Carlsbad, CA, United States) and incubated for 3 h at 20°C followed by an overnight ethanol precipitated. For cDNA synthesis, 1 μL of gene specific primer (10 μM; “*lncCOBRA1* qPCR Reverse set 2”) was added to the ligase reaction and heat treated at 80°C for 3 min followed by 2 min on ice. Reverse transcription was performed with 100 units SuperScript II in 1X First Strand Buffer, 2 mM dNTPs, 10 mM DTT, and 10 units RNaseOUT (Invitrogen; Carlsbad, CA, United States) for 1 h at 42°C, 10 min 50°C, 15 min 70°C, hold at 4°C and store at –20°C overnight.

The first round of PCR was performed using 1X Phusion High-Fidelity PCR Master Mix with HF Buffer (New England BioLabs; Ipswitch, MA, United States) with forward primer “reverse transcription primer (RTP),” and reverse primer “lncCOBRA1 5′ RACE Primer 1” with cDNA diluted 1:5 with the following program: 95°C for 5 min; 30 cycles of 95°C for 30 s, 55°C for 30 s, 72°C for 1 min; 72°C 5 min, hold at 4°C. PCR 2 was performed similarly, but with PCR reaction 1 diluted 1:20 as the template and “Internal RA5 Primer” forward primer and either (A) “lncCOBRA1 qPCR Reverse set 1” or (B) “lncCOBRA1 5’ RACE Primer 2” as the reverse primer. The PCR reaction was then run on a 1% agarose TAE gel with a 1 kb plus DNA ladder (Invitrogen; Carlsbad, CA, United States). All primers are listed in Supplementary Data Set 2.

#### 3′ Rapid Amplification of cDNA Ends

To ligate the 3′ adapter, the 3′ RNA adapter (RA3; 5′-TGGAATTCTCGGGTGCCAAGG -3) was first heated to 70°C for 3 min and snapped cool on ice for 2 min. Eight μL heat-treated 5 μM RA3 was added to 1 μg RNA isolated from 5-day-old Col-0 seedlings and incubated with 200 units T4 RNA Ligase 2, truncated (New England BioLabs; Ipswitch, MA, United States) in 1X T4 RNA Ligase Buffer (New England BioLabs; Ipswitch, MA, United States) for 1 h 15 min at 28°C. As a control, this reaction was also performed in the absence of T4 RNA Ligase 2, truncated (–Lig). The reaction was then ethanol precipitated overnight.

The following day, the precipitated RNA was split in half for reverse transcription ± RT. To 8 μL RNA, 1 μL 10 mM dNTPs and 1 μL RTP was added and incubated for 5 min at 65°C then transferred to ice for 2 min. Reverse transcription was performed with 100 units SuperScriptII (SSII) in 1X First Strand Buffer, 10 mM DTT, 20 units RNaseOUT (Invitrogen; Carlsbad, CA, United States) for 2 min at 25°C, 90 min 42°C, 5 min 95°C, hold at 4°C and store at –20°C overnight. The reaction was also performed without SSII as a control.

The first round of PCR was performed using 1X Phusion High-Fidelity PCR Master Mix with HF Buffer (New England BioLabs; Ipswitch, MA, United States) with cDNA diluted 1:10 in water, and forward primer “RTP” and reverse primer “Illumina RNA index primer 35” with the following program: 95°C for 5 min; 30 cycles of 95°C for 30 s, 55°C for 30 s, 72°C for 1 min; 72°C 5 min, hold at 4°C. PCR 2 was performed similarly, but with PCR reaction 1 diluted 1:20 as the template and “lncCOBRA1 3’ RACE Primer 1” forward primer and “RNA primer index universal” as the reverse primer. The PCR reaction was then run on a 1% agarose TAE gel with a 1 kb plus DNA ladder (Invitrogen; Carlsbad, CA, United States), excised and gel extracted using the Monarch DNA Gel Extraction Kit following the manufacturers’ instructions (New England BioLabs; Ipswitch, MA, United States).

The purified PCR reaction was then A-tailed with 15 units Klenow Fragment (3′–5′ exo-) (New England BioLabs; Ipswitch, MA, United States) in 1X NEB Buffer 2 (New England BioLabs; Ipswitch, MA, United States), and 0.1 mM dATP for 30 min at 37°C. The reaction was then cleaned up using Zymo ChIP DNA Clean & Concentrator following the manufacturers’ instructions (Zymo Research; Irvine, CA, United States). The resulting PCR reaction was then cloned into pGEM T-Vector system and selected for using the XGal/IPTG system (Promega, Madison, WI, United States). Sanger sequencing was performed at the University of Pennsylvania Genomic Analysis Core with the SP6 promoter/primer. All primers are listed in Supplementary Data Set 2.

### Denaturing RNA Gel

Gel was performed using NorthernMax reagents (Invitrogen; Carlsbad, CA, United States). Ten μg of total RNA for each genotype was added to appropriate amount of 3X NorthernMax Formaldehyde Loading dye, boiled at 65°C for 15 min and flash cooled on ice. 0.5 μL of ethidium bromide (10 mg/mL) was added to each sample and loaded onto a 1.5% NorthernMax denaturing agarose gel and run for ∼3 h at 100 V. Gel was visualized by UV.

### Germination

For germination experiments, seeds of Col-0, *lnccobra1-1*, *lnccobra1-2*, and *lncCOBRA1/lnccobra1-1* were sterilized in 100% ethanol for 1-min followed by a 10-min wash with 30% Clorox and 0.01% Tween-20 and washed 5X with sterilized water. Seeds were then plated on 1/2 MS agar plates with 1% sucrose and 0.8% Phytoblend and stratified by cold treating at 4°C for 48 h and placed in growth chambers. Two days after transfer to growth chambers, the number of seeds that that displayed cotyledons entirely emerged from the seed coat were counted. Plates were then allowed to grow for 3 more days and 5-day-old seedlings were collected to measure *lncCOBRA1* abundance.

### Leaf Initiation Rate

Col-0, *lnccobra1-1*, *lnccobra1-2*, and *lncCOBRA1/lnccobra1-1* were grown in soil as described above. Every day at ∼11 AM the presence of leaf primordia was examined. Leaf initiation was measured when the leaf primordia was visible to the eye (∼0.5 mm). After 3 weeks, plants were weighed for fresh weight measurements. To measure plant size, 3-week-old plants were taped flat on paper, scanned, and analyzed using ImageJ as follows. Scanned images were first converted to 8-bit and processed into a binary image such that any plant tissue was converted to white and background became black. Threshold was set using default settings, inverted, and the “particles” (plants) perimeter and area measured. Area of leaf 3 was selected by hard and measured.

### Chromatin Isolation by RNA Purification

#### Probe Design, Crosslinking and Chromatin Isolation

Chromatin isolation by RNA purification probes were designed using the Stellaris probe website^4^ with a 3′ Biotin TEG. 5-day-old Col-0 and *lnccobra1-2* seedlings were crosslinked in PBS with 1% formaldehyde (v/v) (Sigma-Aldrich; St. Louis, MO, United States) added and placed under vacuum for 10 min, followed by a 5-min quench with 125 mM Glycine under vacuum. Crosslinked tissue was then washed five times in distilled, deionized water, patted dry with paper towels, flash frozen in liquid nitrogen, and stored at –80°C until further processing. Chromatin from 6 g of 5-day-old Col-0 and *lnccobra1-2* crosslinked seedlings (3 g scrambled probes and 3 g *lncCOBRA1* probes) was isolated as previously described (Do et al., 2019). All probes are listed in Supplementary Data Set 2.

#### Bead Preparation

Pierce High Capacity Streptavadin Agarose beads (Thermo Fisher Scientific; Carlsbad, CA, United States) were first chemically treated to protect the streptavidin from tryptic proteolysis in preparation for mass spectrometry to reduce streptavidin signal as previously described (Barshop et al., 2019).

#### Chromatin Isolation by RNA Purification

Chromatin isolation by RNA purification was performed as previously described (Chu et al., 2011, 2012, 2015), with several modifications. Modified Pierce High Capacity Streptavadin Agarose beads (Thermo Fisher Scientific; Carlsbad, CA, United States) were first washed twice and resuspended in nuclei lysis buffer (50 mM Tris-HCl pH = 8.0, 10 mM EDTA, 1% SDS) supplemented with cOmplete Protease Inhibitor Cocktail (Sigma, St Louis, MO, United States) and RNaseOUT (Invitrogen, Carlsbad, CA, United States). Chromatin lysates were then pre-cleared with 30 μL modified beads for 30 min with mixing in a 37°C hybridization oven with rotation. After pre-clearing, samples were centrifuged twice at 3000 RPM for 5 min at room temperature (RT) to thoroughly remove any beads, and 10% of the sample was removed for both RNA input and protein input. The lysates were then split into a scrambled and *lncCOBRA1* probe sample and 2X Hybridization buffer was added (750 mM NaCl, 1% SDS, 50 mM Tris-HCl pH = 7.5, 1 mM EDTA, 15% Formamide) supplemented with PMSF (100 μL/10 mL), RNaseOUT (5 μL/10 mL; Invitrogen, Carlsbad, CA, United States), and cOmplete Protease Inhibitor Cocktail (Sigma, St Louis, MO, United States). 100 pmol of probes were then added per 1 mL chromatin (i.e., 1.67 μL for each of the 6 probes used for *lncCOBRA1*) and the samples incubated in a 37°C hybridization oven with rotation.

After 5 h, 100 μL of modified beads were added to each tube and incubated in a 37°C hybridization oven with rotation for another 2 h. Samples were then centrifuged for 5 min at 3000 RPM, supernatant removed, and resuspended in 1 mL wash buffer (2S SSC, 0.5% SDS) pre-warmed to 37°C and incubated in a 37°C hybridization oven with rotation for another 30 min. Samples were washed for a total of four washes. After the last spin, samples are resuspended in 1 mL wash buffer and 150 μL removed for RNA isolation and the remaining 850 μL used for mass spectrometry.

#### RNA Isolation

RNA isolation was performed using a modified version of a previously published protocol (Desvoyes et al., 2018). RNA samples were centrifuged at 3000 RPM for 5 min and resuspended in 400 μL RNA proteinase K buffer (PK Buffer; 100 mM NaCl, 10 mM Tris-HCl pH = 7.5, 1 mM EDTA, 0.5% SDS) and 390 μL PK buffer was added to RNA input samples. To reverse crosslinks, NaCl was added to a final concentration of 200 mM (add 8 μL 5M NaCl) and incubated at 65°C overnight. The following day 16 μL 1M Tris-HCl pH = 6.8, 8 μL 0.5 M EDTA and 2 μL proteinase K (Denville Scientific; Metuchen, NJ, United States) was added and incubated at 37°C for 2 h with rotation to remove proteins. Samples were then added to 700 μL Qiazol (Qiagen; Valencia, CA, United States), and RNA isolated as described above. All primers are listed in Supplementary Data Set 2.

#### ChIRP-MS qPCR Validation

qPCR was performed as described above with the following exceptions. A standard curve for all primer sets was generated using serial dilutions of genomic DNA. “Copy number” of each transcript was calculated, and normalized by the average *C*_*T*_ value for three technical replicates of *U6* for each sample. The normalized values were then used to calculate fold enrichment relative to Col-0 input.

#### Mass Spectrometry Sample Preparation and Acquisition

Protein samples were centrifuged at 3000 RPM for 5 min, supernatant removed, and the beads were wash three times with 100 mM NH_4_HCO_3_, and ultimately resuspended in 400 μL 100 mM NH_4_HCO_3_ supplemented with 200 mM NaCl and incubated overnight at 65°C to reverse crosslinks. The next day the samples were flash frozen in liquid nitrogen and stored at –80°C until processing. Samples were thawed on ice and resuspended in an appropriate volume of the resuspension buffer [50 mM SDS and 50 mM triethylammonium bicarbonate (TEAB) final concentrations] and reduced with final 10 mM DTT (US Biological, Salem, MA, United States) for 30 min at 30 °C, followed by alkylation with final 50 mM iodoacetamide (Sigma, St Louis, MO, United States) for 30 min at 30 °C. The samples were processed using an S-Trap™ column according to the protocol recommended by the supplier (Protifi; Farmingdale, NY, United States; C02-mini): loaded onto the column and digested with trypsin (Thermo Fisher Scientific; Carlsbad, CA, United States) in 1:10 (w/w) enzyme/protein ratio for 1 h at 47 °C.

Peptides eluted from this column were vacuum-dried and resuspended with LC-MS grade water containing 0.1% (v/v) TFA for mass spectrometry analysis. Each sample was analyzed by a Q-Exactive HF mass spectrometer (Thermo Fisher Scientific; Carlsbad, CA, United States) coupled to a Dionex Ultimate 3000 UHPLC system (Thermo Fisher Scientific; Carlsbad, CA, United States) equipped with an in-house made 15 cm long fused silica capillary column (75 μm ID), packed with reversed−phase Repro−Sil Pur C18−AQ 2.4 μm resin (Dr. Maisch; GmbH, Ammerbuch, Germany) column. Elution was performed using a gradient from 5 to 35% B (50 min), followed by 90% B (10 min), and re-equilibration from 90 to 5% B (5 min) with a flow rate of 400 nL/min (mobile phase A: water with 0.1% formic acid; mobile phase B: 80% acetonitrile with 0.1% formic acid). Data were acquired in data-dependent MS/MS mode. Full scan MS settings were: mass range 200-1500 m/z, resolution 120,000; MS1 AGC target 1E6; MS1 Maximum IT 100. MS/MS settings were: resolution 30,000; AGC target 5E4; MS2 Maximum IT 200 ms; fragmentation was enforced by higher-energy collisional dissociation with stepped collision energy of 25, 27, 30; loop count top 15; isolation window 1.4; fixed first mass 120; MS2 Minimum AGC target 2E3; charge exclusion: unassigned, 1, 7, 8, and >8; peptide match preferred; exclude isotope on; dynamic exclusion 45 s.

#### Mass Spectrometry Data Analysis

The acquired data were processed *via* Proteome Discoverer 2.4 with the default QExactive Precursor Quant and LFQ MPS with SequestHT and Percolator processing template and Comprehensive Enhanced Annotation LFQ and Precursor Quant consensus template with the following parameters. The spectra match with peptide sequence was performed with SequestHT with contaminants.fasta from MaxQuant and *Arabidopsis thaliana* fasta 2019.04 release, full tryptic digestion, maximum missed cleavage 3, peptide length between 6 and 144, MS1 mass tolerance 10 ppm, MS2 mass tolerance 0.02 Da, dynamic modification with oxidation on methionine, acetylation and methionine loss on protein N-terminal, static modification with carbamidomethyl on cysteine. The protein inference and identification validation was performed with Percolator with a 1% false discovery rate (FDR) cut off. Normalization was performed by total peptide amount and scaling mode was set to on all average. Protein Abundance was peptide summed with the top three most abundant peptides for each protein.

Proteins were first filtered such that only proteins with abundance scores in at least two biological replicates of the pulldown with the *lncCOBRA1* probes in Col-0 background were considered. Protein abundance in *lncCOBRA1* pulldown was then normalized by the average protein abundance identified using the scrambled probes (*lncCOBRA1*/scrambled) and only proteins that were enriched with the *lncCOBRA1* probes compared to the scrambled sequence probes were examined further (*COBRA1*/scrambled > 1). Enrichment of Col-0 over *lnccobra1-2* background was then calculated as the log_2_[Col-0/*lnccobra1-2*] and proteins enriched over 1-fold were classified as *lncCOBRA1*-interacting and used for future analyses. We also examined proteins that were present in at least 2 biological replicates of the *lncCOBRA1* pulldown in Col-0 tissue, but absent from scrambled and in 0 or 1 biological replicate of the *lncCOBRA1* pulldown in the *lnccobra1-2* background. Since no protein abundances were found in the scrambled, a fold enrichment could not be calculated.

### Protein–Protein Interaction Network

STRING^5^ was used to generate the protein-protein interaction network with medium stringency and clustered into five clusters by the k-means clustering algorithm provided. PPI enrichment was calculated by the STRING program (Szklarczyk et al., 2019).

## Significance Statement

Long non-coding RNAs (lncRNAs) are an important yet understudied class of molecules in all eukaryotic organisms. While thousands of lncRNAs have been identified, only a handful have described functions. In this work, the authors describe a previously uncharacterized and highly conserved lncRNA that contains two snoRNA domains, and functions to affect Arabidopsis germination and growth. Overall, the authors describe sno-lncRNAs for the first time in a non-mammalian system and demonstrate novel mechanisms of lncRNA function in Arabidopsis.

## Data Availability Statement

The datasets presented in this study can be found in online repositories. The names of the repository/repositories and accession number(s) can be found below: The ProteomeXchange Consortium via the PRIDE (1) partner repository with the dataset identifier PXD033707.

## Author Contributions

MCK and BAG conceived the study and wrote the manuscript with assistance from all authors. MCK, HJK, KRP, and BAG designed the experiments. MCK, HJK, and KRP performed experiments and analyzed the data. All authors have read and approved the manuscript for publication.

## Conflict of Interest

EL was employed by CyVerse Inc. The remaining authors declare that the research was conducted in the absence of any commercial or financial relationships that could be construed as a potential conflict of interest.

## Publisher’s Note

All claims expressed in this article are solely those of the authors and do not necessarily represent those of their affiliated organizations, or those of the publisher, the editors and the reviewers. Any product that may be evaluated in this article, or claim that may be made by its manufacturer, is not guaranteed or endorsed by the publisher.

## Footnotes

1
https://www.addgene.org

2
https://crispr.dbcls.jp

3
https://genomevolution.org/CoGe/CoGeBlast.pl

4
https://www.biosearchtech.com/support/tools/design-software/chirp-probe-designer

5
https://string-db.org/cgi/input.pl?sessionId=QFOfEIHPOaYj&input_page_show_search=on

## Supplementary Material

Supplementary Figure 1 Identification of highly conserved, protein-bound lincRNAs in the nuclei from 10-day-old seedlings. **(A)** Flowchart diagram of identification of *lncCOBRA* transcripts from protein interaction profile sequencing (PIP-seq) in the nuclei from 10-day-old seedlings (Gosai et al., 2015). **(B)** Abundance of all *lncCOBRA* transcripts during germination. Abundance is relative to dry seed after harvest. Data was provided in Narsai et al. (2017). Asterisk denotes lincRNAs with snoRNAs annotated within them. Raw values are listed in Supplementary Data Set 3. **(C)** eFP browser views of abundance of *lncCOBRA1*, *lncCOBRA3*, and *lncCOBRA5* during germination (Klepikova et al., 2016). **(D)** Abundance of *lncCOBRA1* early seedling development using primer set 2 as measured by qPCR. Abundance is normalized by *UBC9* and *UBC10* and is relative to 7-day-old seedlings. ^***^ Denotes *p*-value < 0.001, Wilcoxon *t*-test. **(E)** Abundance of *lncCOBRA1* using primer set 2 as measured by qPCR. Abundance is normalized by *UBC9* and *UBC10* and is relative to siliques seedlings. **(F)** Gene model of *lncCOBRA3* (*AT3G03445*) and nearby snoRNAs (*AT3G03445*, *AT3G03425*, and *AT3G03415*). **(G)** Diagram of *lncCOBRA1* representing the location of the two sets of primers used for qPCR.

Supplementary Figure 2 *lncCOBRA1* is highly conserved. **(A)** Percent nucleotide identity in *Brassica rapa, Camelina sativa, Capsella rubella*, and *Eutrema salsugineum* in the 500 nt up- and downstream of *AT1G05917* (sno-*COBRA1A*). Calculated by Geneious Prime (Geneious | Bioinformatics Solutions for the Analysis of Molecular Sequence Data, 2019). **(B)** Comparison between the sequence of sno-*COBRA1A* and sno-*COBRA1B* and their human homologs. Performed using blastn suite from the NCBI aligning two of more sequences (Zhang et al., 2000).

Supplementary Figure 3 *lncCOBRA1* is transcribed as a longer transcript with conserved promoter elements. **(A)** Diagram of *lncCOBRA1* representing the location of the RT-PCR primers (green arrows). **(B)** RT-PCR in cDNA from seeds 1- and 2-day-after soaking in water, and 2-, 3-, 4-, and 5-day-old seedlings. Water is a negative control and genomic Col-0 DNA was used as a positive control. Ladder is 1 kb plus. **(C)** Diagram of the promoter region and conserved elements. Yellow boxes indicate Site II elements, and the purple box represents a Telo-box, together forming a *Telo*SII element. The orange box represents a conserved non-coding sequence (CNS) and the green box represents a TATA-box.

Supplementary Figure 4 Loss of *lncCOBRA1* slightly affects rRNA abundance. **(A)** Gel image confirming large deletion caused by two guide RNAs targeted to the 5′ end of *lncCOBRA1*. *GAPDH* is a positive control. **(B)** Diagram of *lncCOBRA1* representing the location of the T-DNA insertion in *lnccobra1-1*, location of guide RNAs and actual sites of deletion (represented by red triangles) for *lnccobra1-2*. **(C)** Relative abundance of *COBRA1* in Col-0, *lnccobra1-1*, and *lnccobra1-1/lncCOBRA1pro:lncCOBRA1* using primer set 2 as measured by qPCR. Abundance is normalized by *UBC9* and *UBC10* and is relative to Col-0. ^***^ Denotes *p*-value < 0.001; Wilcoxon *t*-test. *N* = 3. Error bars represent SEM. **(D)** Relative abundance of *lncCOBRA1* (set 1), *5.8S*, *18S*, and *25S rRNA* in Col-0, *lnccobra1-1, lnccobra1-2*, and *lnccobra1-1/lncCOBRA1pro:lncCOBRA1*. Abundance is normalized by *UBC9* and *UBC10* and is relative to Col-0. ^***^ Denotes *p*-value < 0.001; Wilcoxon *t*-test. *N* = 3. Error bars represent SEM. **(E)** Total RNA isolated from Col-0, *lnccobra1-1, lnccobra1-2*, and *lnccobra1-1/lncCOBRA1pro:lncCOBRA1* on a 1.5% denaturing agarose gel.

Supplementary Figure 5 Loss of *lncCOBRA1* results in smaller plants. **(A)** Representative images generated from ImageJ to measure perimeter of 3-week-old plants. Images on top and bottom are the same plants, with the top being used for perimeter measurements and the bottom used for area measurements. **(B)** Leaf area of leaf three measured by ImageJ. * Denotes *p*-value < 0.05; Wilcoxon *t*-test.

Supplementary Figure 6 *lncCOBRA1* does not interact with rRNAs. **(A)** Relative abundance of *5.8S rRNA*, *18S rRNA*, and *25S rRNA* in ChIRP-MS experiments. Abundance is normalized by *U6* and relative to Col-0 input. Error bars represent SEM. ns and ^**^, denotes *p*-value > 0.05 and <0.01, respectively; Wilcoxon *t*-test. *N* = 3. **(B)** Overlap between *lncCOBRA1*-interacting proteins and proteins classified as RBPs in an RNA binding proteome capture experiment in Arabidopsis leaves. ^***^ Denotes *p*-value < 0.001; Hypergeometric test. **(C)** Gene ontology enrichment analysis for biological function using Plant Regulomics (Ran et al., 2020) for *COBRA1*-interacting proteins. Size of circles represents –log_10_ (*p*-value).

Supplementary Figure 7 *lncCOBRA1*-interactome. **(A)** Protein–protein interaction (PPI) network for *lncCOBRA1*-interacting proteins. Proteins were clustered into five clusters by k-means clustering. Thickness of lines connecting notes indicates the confidence of that protein-protein interaction. Dotted line indicates interaction with a different cluster. **(B)** Gene ontology enrichment for proteins in each cluster (Huang et al., 2009).

## Abbreviations

lncRNA: long non-coding RNA
lincRNA: long intergenic non-coding RNA
snoRNA: small nucleolar RNA
nt: nucleotide
RBP: RNA binding protein.
