# Supplementary figures and images for: A Conserved Long Intergenic Non-coding RNA Containing snoRNA Sequences, lncCOBRA1, Affects Arabidopsis Germination and Development
Source: Front Plant Sci. 2022 May 25;13:906603. doi: 10.3389/fpls.2022.906603 (PMC9175010; doi:10.3389/fpls.2022.906603)

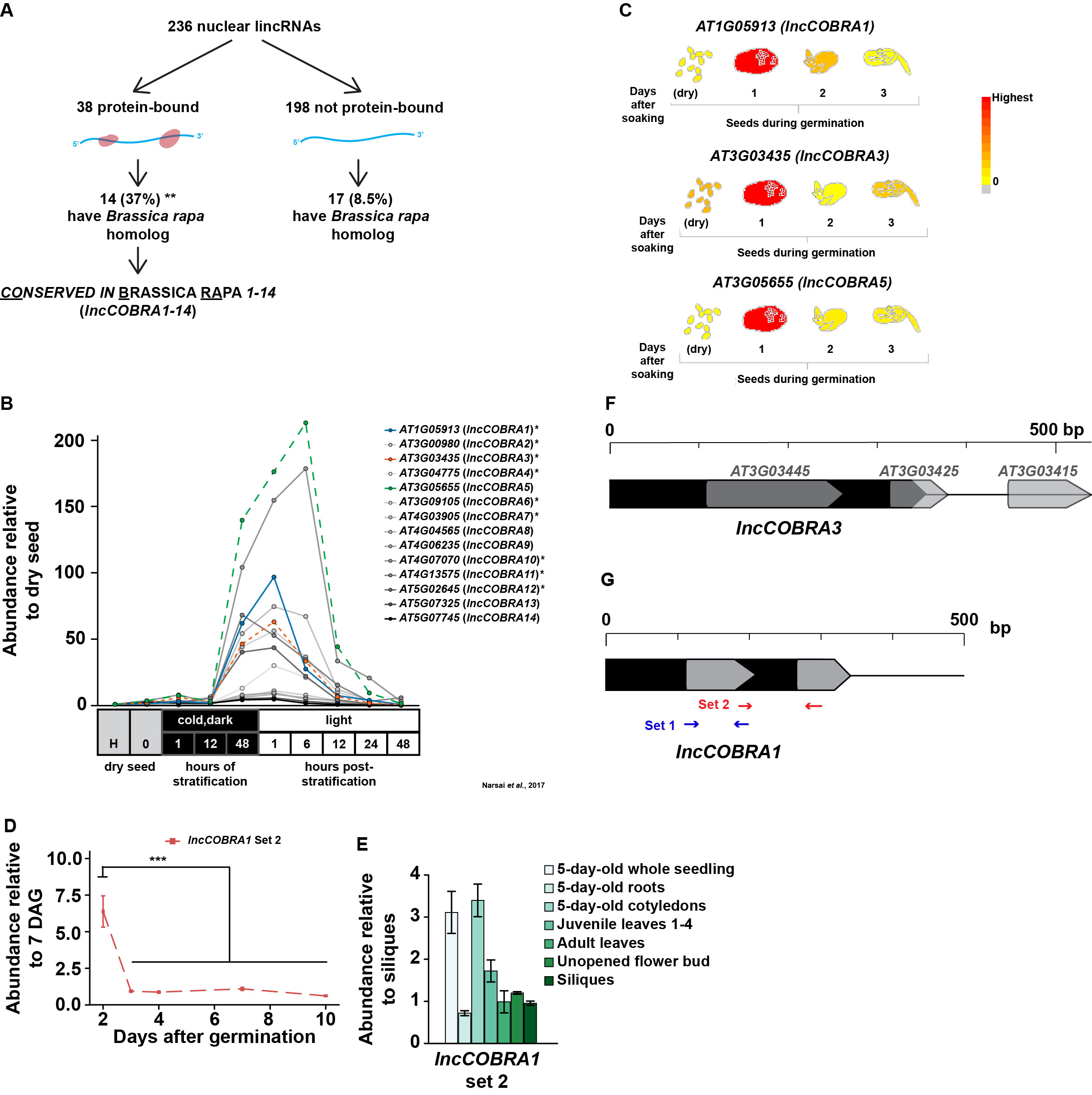

Supplement: Supplementary Figure 1 — Identification of highly conserved, protein-bound lincRNAs in the nuclei from 10-day-old seedlings. (A) Flowchart diagram of identification of lncCOBRA transcripts from protein interaction profile sequencing (PIP-seq) in the nuclei from 10-day-old seedlings (Gosai et al., 2015). (B) Abundance of all lncCOBRA transcripts during germination. Abundance is relative to dry seed after harvest. Data was provided in Narsai et al. (2017). Asterisk denotes lincRNAs with snoRNAs annotated within them. Raw values are listed in Supplementary Data Set 3. (C) eFP browser views of abundance of lncCOBRA1, lncCOBRA3, and lncCOBRA5 during germination (Klepikova et al., 2016). (D) Abundance of lncCOBRA1 early seedling development using primer set 2 as measured by qPCR. Abundance is normalized by UBC9 and UBC10 and is relative to 7-day-old seedlings. *** Denotes p-value < 0.001, Wilcoxon t-test. (E) Abundance of lncCOBRA1 using primer set 2 as measured by qPCR. Abundance is normalized by UBC9 and UBC10 and is relative to siliques seedlings. (F) Gene model of lncCOBRA3 (AT3G03445) and nearby snoRNAs (AT3G03445, AT3G03425, and AT3G03415). (G) Diagram of lncCOBRA1 representing the location of the two sets of primers used for qPCR. [file Image_1.JPEG]

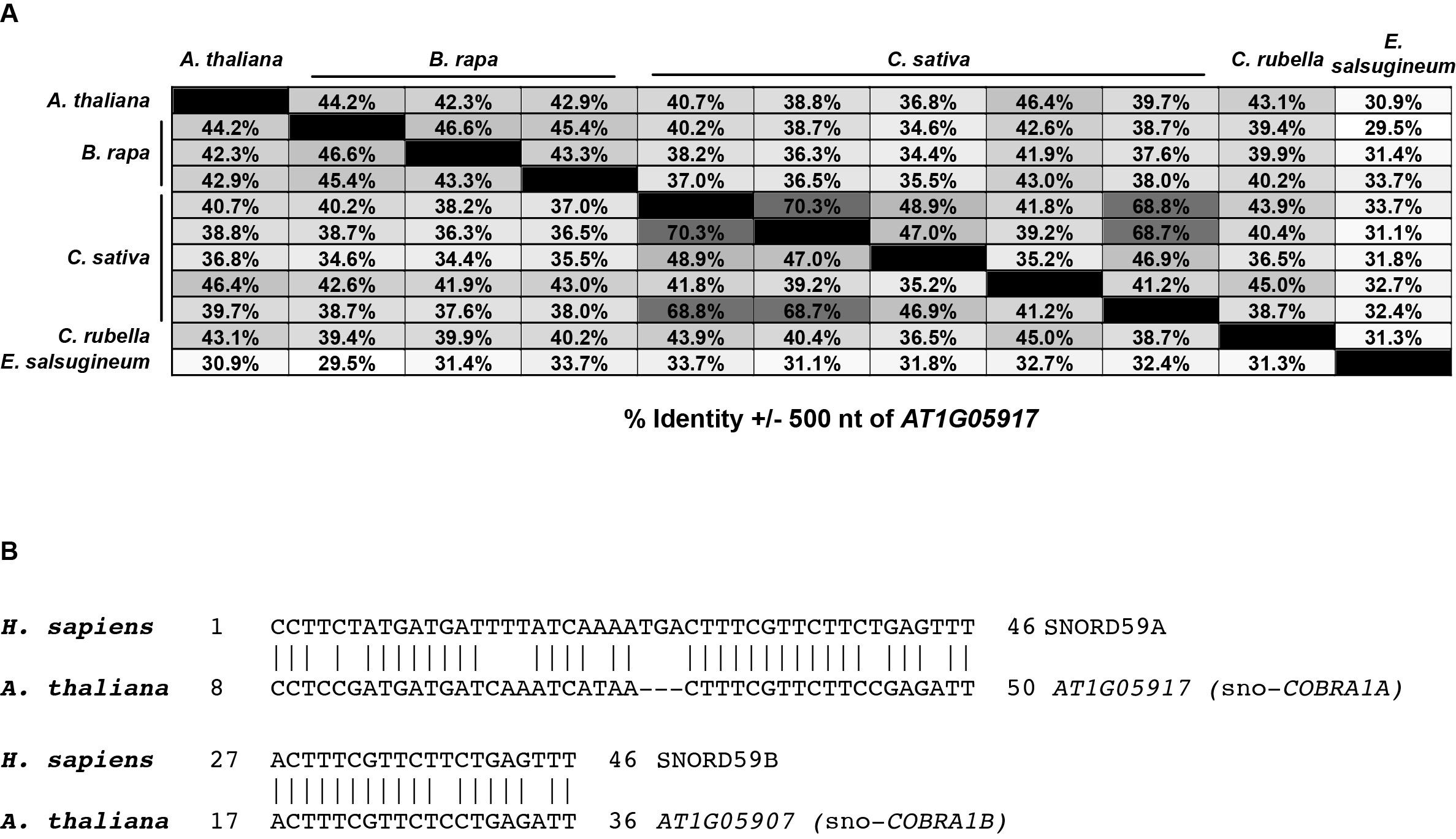

Supplement: Supplementary Figure 2 — lncCOBRA1 is highly conserved. (A) Percent nucleotide identity in Brassica rapa, Camelina sativa, Capsella rubella, and Eutrema salsugineum in the 500 nt up- and downstream of AT1G05917 (sno-COBRA1A). Calculated by Geneious Prime (Geneious | Bioinformatics Solutions for the Analysis of Molecular Sequence Data, 2019). (B) Comparison between the sequence of sno-COBRA1A and sno-COBRA1B and their human homologs. Performed using blastn suite from the NCBI aligning two of more sequences (Zhang et al., 2000). [file Image_2.JPEG]

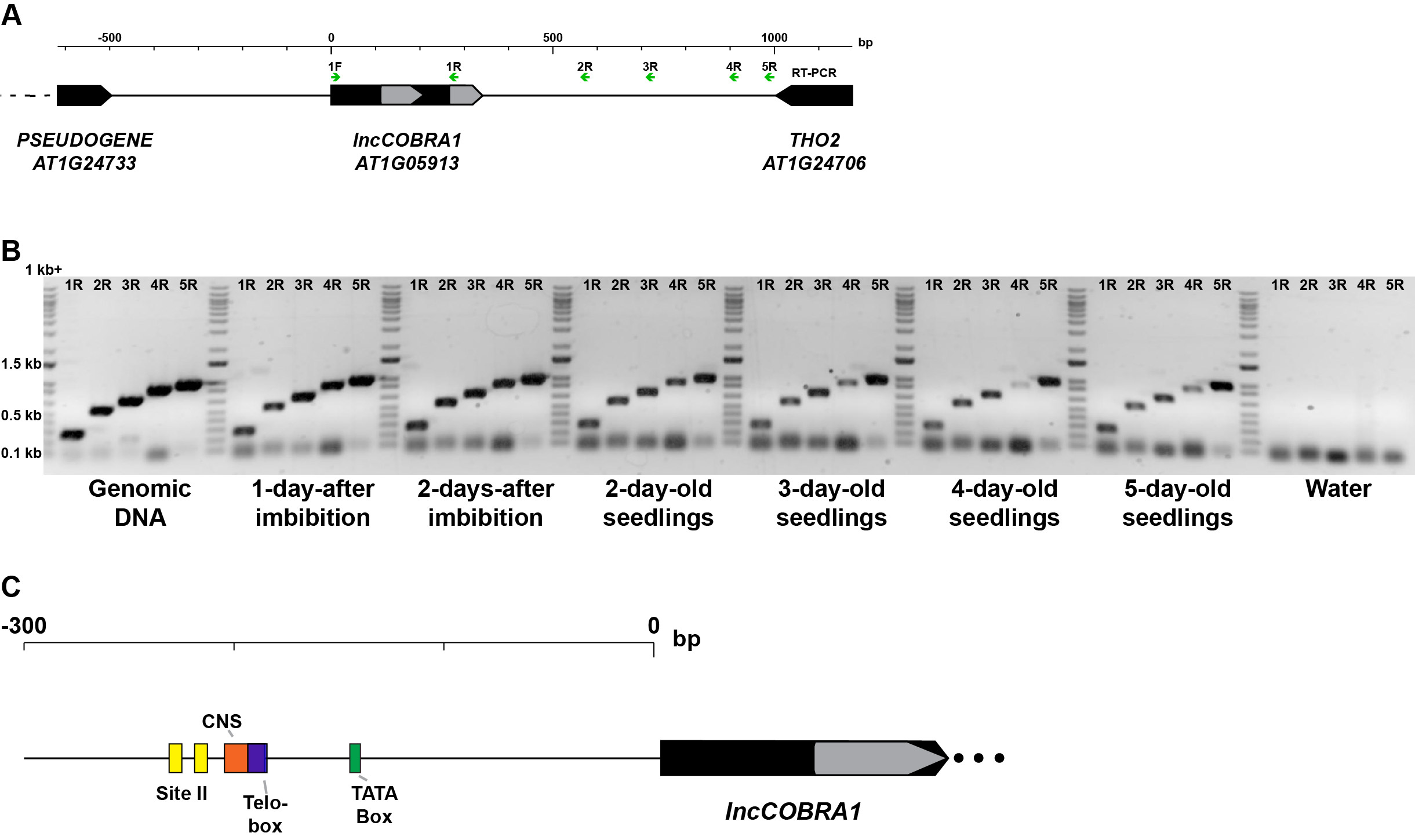

Supplement: Supplementary Figure 3 — lncCOBRA1 is transcribed as a longer transcript with conserved promoter elements. (A) Diagram of lncCOBRA1 representing the location of the RT-PCR primers (green arrows). (B) RT-PCR in cDNA from seeds 1- and 2-day-after soaking in water, and 2-, 3-, 4-, and 5-day-old seedlings. Water is a negative control and genomic Col-0 DNA was used as a positive control. Ladder is 1 kb plus. (C) Diagram of the promoter region and conserved elements. Yellow boxes indicate Site II elements, and the purple box represents a Telo-box, together forming a TeloSII element. The orange box represents a conserved non-coding sequence (CNS) and the green box represents a TATA-box. [file Image_3.JPEG]

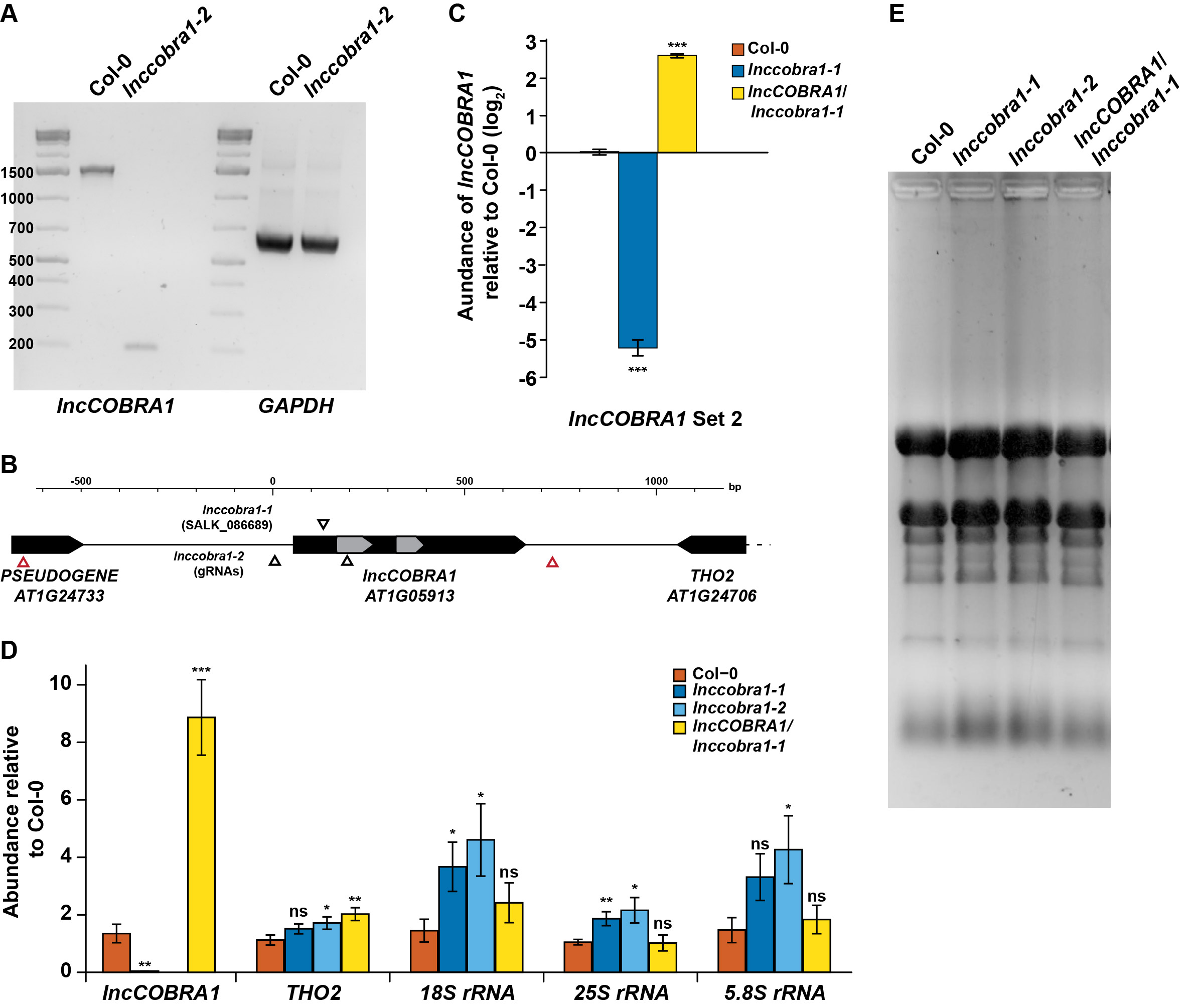

Supplement: Supplementary Figure 4 — Loss of lncCOBRA1 slightly affects rRNA abundance. (A) Gel image confirming large deletion caused by two guide RNAs targeted to the 5′ end of lncCOBRA1. GAPDH is a positive control. (B) Diagram of lncCOBRA1 representing the location of the T-DNA insertion in lnccobra1-1, location of guide RNAs and actual sites of deletion (represented by red triangles) for lnccobra1-2. (C) Relative abundance of COBRA1 in Col-0, lnccobra1-1, and lnccobra1-1/lncCOBRA1pro:lncCOBRA1 using primer set 2 as measured by qPCR. Abundance is normalized by UBC9 and UBC10 and is relative to Col-0. *** Denotes p-value < 0.001; Wilcoxon t-test. N = 3. Error bars represent SEM. (D) Relative abundance of lncCOBRA1 (set 1), 5.8S, 18S, and 25S rRNA in Col-0, lnccobra1-1, lnccobra1-2, and lnccobra1-1/lncCOBRA1pro:lncCOBRA1. Abundance is normalized by UBC9 and UBC10 and is relative to Col-0. *** Denotes p-value < 0.001; Wilcoxon t-test. N = 3. Error bars represent SEM. (E) Total RNA isolated from Col-0, lnccobra1-1, lnccobra1-2, and lnccobra1-1/lncCOBRA1pro:lncCOBRA1 on a 1.5% denaturing agarose gel. [file Image_4.JPEG]

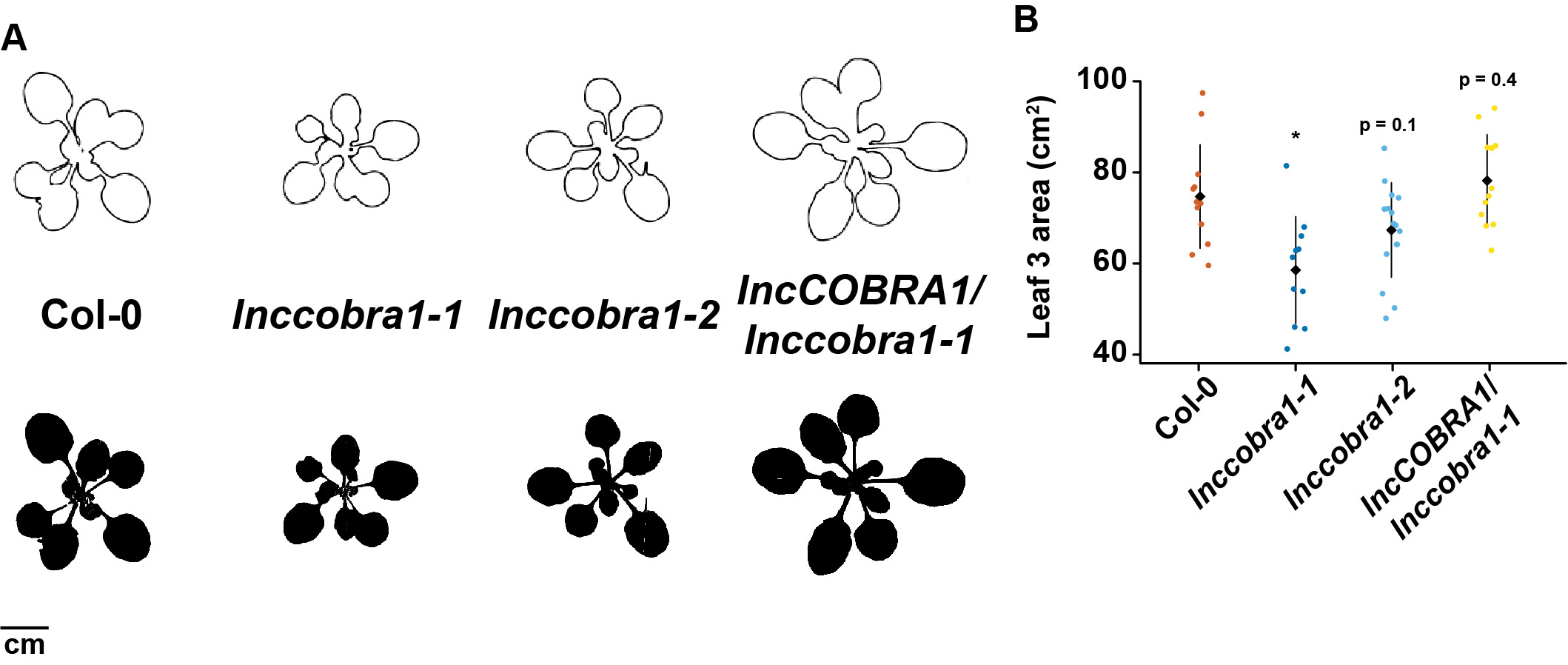

Supplement: Supplementary Figure 5 — Loss of lncCOBRA1 results in smaller plants. (A) Representative images generated from ImageJ to measure perimeter of 3-week-old plants. Images on top and bottom are the same plants, with the top being used for perimeter measurements and the bottom used for area measurements. (B) Leaf area of leaf three measured by ImageJ. * Denotes p-value < 0.05; Wilcoxon t-test. [file Image_5.JPEG]

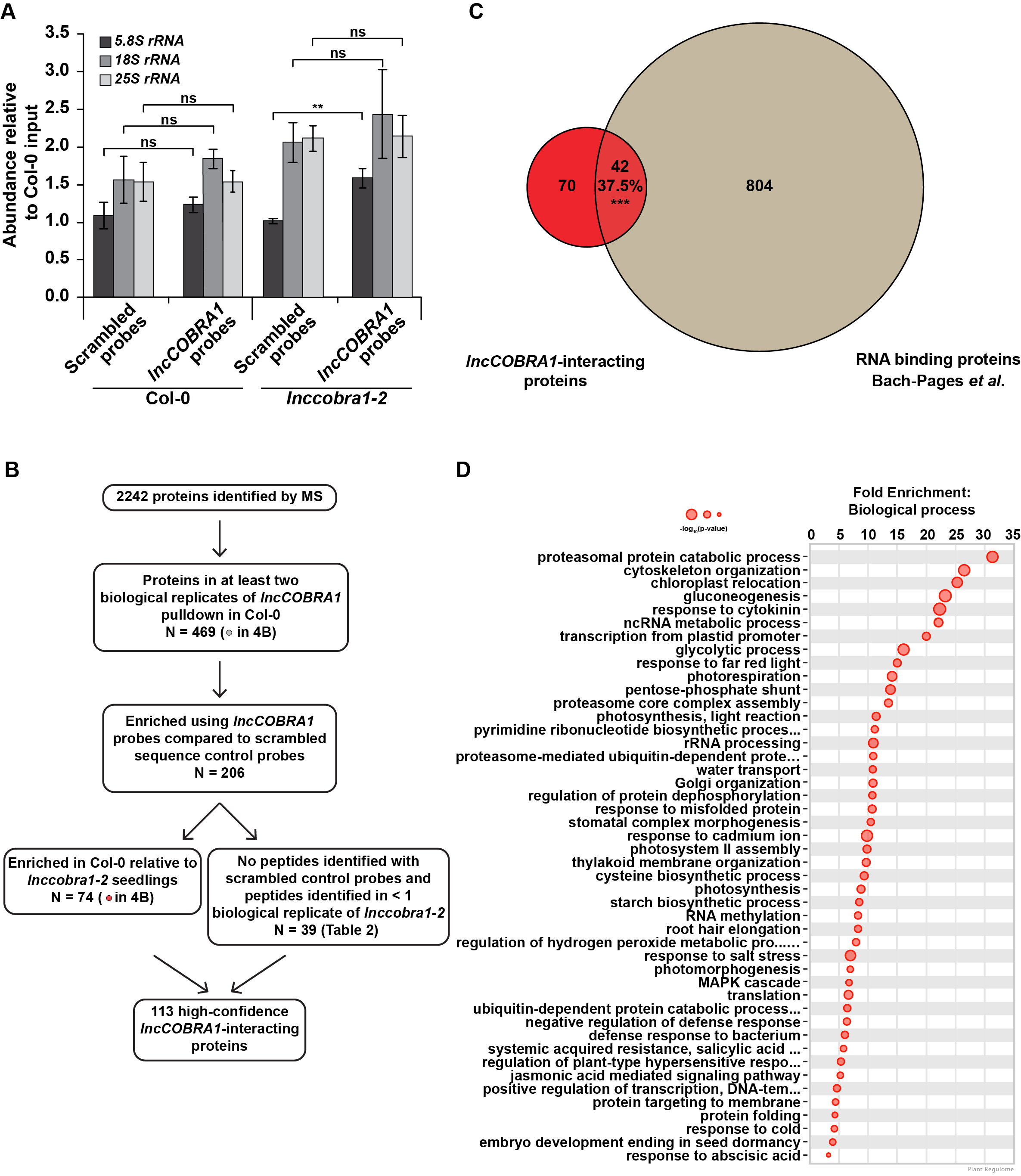

Supplement: Supplementary Figure 6 — lncCOBRA1 does not interact with rRNAs. (A) Relative abundance of 5.8S rRNA, 18S rRNA, and 25S rRNA in ChIRP-MS experiments. Abundance is normalized by U6 and relative to Col-0 input. Error bars represent SEM. ns and **, denotes p-value > 0.05 and <0.01, respectively; Wilcoxon t-test. N = 3. (B) Overlap between lncCOBRA1-interacting proteins and proteins classified as RBPs in an RNA binding proteome capture experiment in Arabidopsis leaves. *** Denotes p-value < 0.001; Hypergeometric test. (C) Gene ontology enrichment analysis for biological function using Plant Regulomics (Ran et al., 2020) for COBRA1-interacting proteins. Size of circles represents –log10 (p-value). [file Image_6.JPEG]

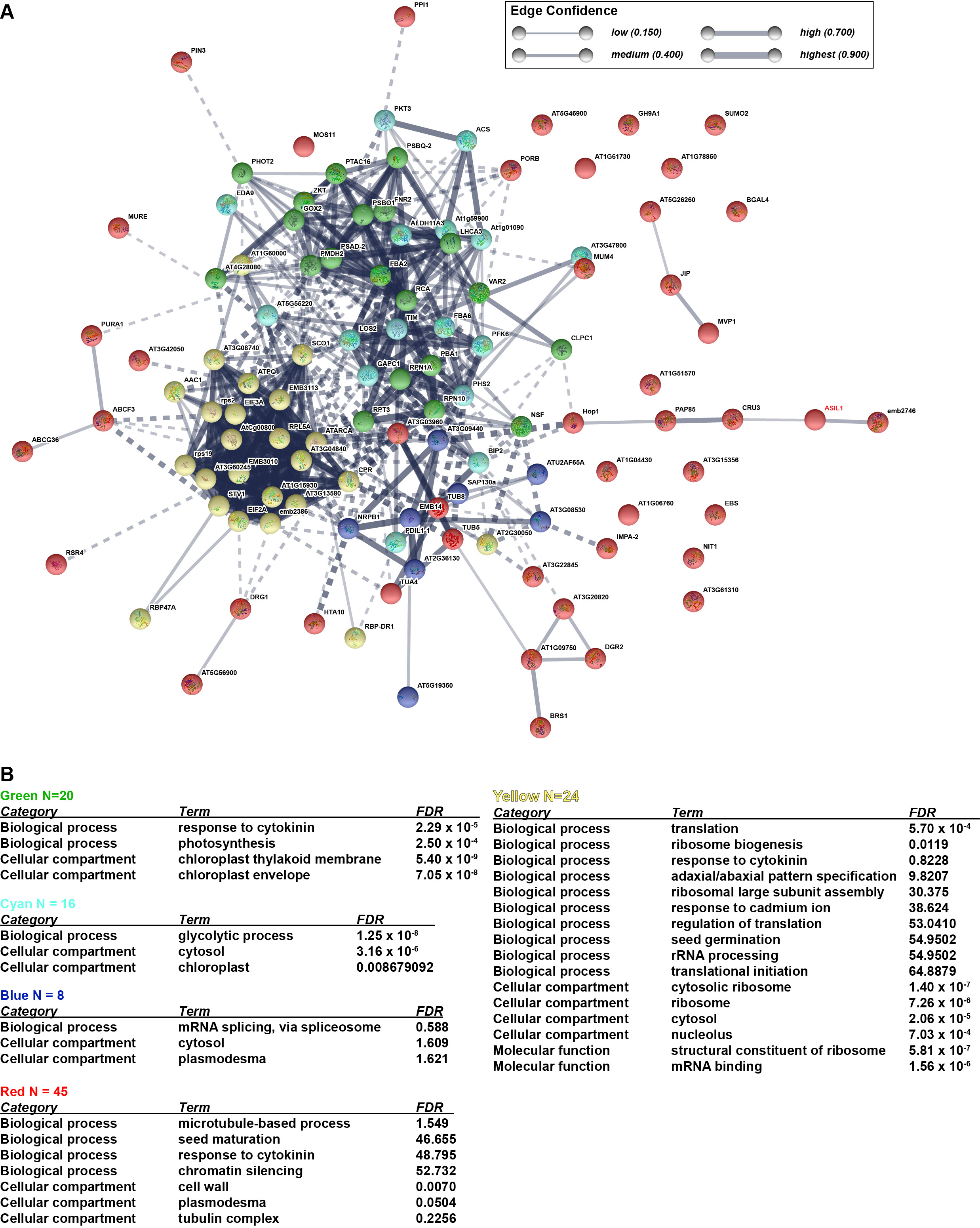

Supplement: Supplementary Figure 7 — lncCOBRA1-interactome. (A) Protein–protein interaction (PPI) network for lncCOBRA1-interacting proteins. Proteins were clustered into five clusters by k-means clustering. Thickness of lines connecting notes indicates the confidence of that protein-protein interaction. Dotted line indicates interaction with a different cluster. (B) Gene ontology enrichment for proteins in each cluster (Huang et al., 2009). [file Image_7.JPEG]
